# Supplementary material for: T-Cell Receptor Sequences Identify Combined Coxsackievirus–Streptococci Infections as Triggers for Autoimmune Myocarditis and Coxsackievirus–Clostridia Infections for Type 1 Diabetes
Source: Int J Mol Sci. 2024 Feb 1;25(3):1797. doi: 10.3390/ijms25031797 (PMC10855694; doi:10.3390/ijms25031797)
Supplement: Supplementary file 1 [file ijms-25-01797-s001.zip › ijms-2736363-supplementary.pdf]

Similarity Results for Diabetes TCR

A1: CASRDRGGGEKLFF

polyprotein, partial [Coxsackievirus B1] UEU84431.1
```
Query  1    CASRDR  6
            CASRDR
Sbjct  913  CASRDR  918
```

polyprotein [Coxsackievirus B4] QQL13732.1
```
Query  1    CASRDR  6
            CASR+R
Sbjct  914  CASRNR  919
```

polyprotein, partial [Coxsackievirus A9] AAF27783.1
```
Query  1    CASRDR  6
            CASR+R
Sbjct  103  CASRNR  108
```

thioesterase family protein [Clostridium tetani] WP_130004577.1
```
Query  9   GEKLFF  14
           GEKLFF
Sbjct  91  GEKLFF  96
```

```
>>sp|P06213|INSR_HUMAN Insulin receptor OS=Homo sapiens
       10
EMBOSS GEKLFF
       :..:::
sp|P06 GSRLFF

Waterman-Eggert score: 21;  14.1 bits; E(1) <  0.68
55.6% identity (66.7% similar) in 9 aa overlap (6-14:1343-1351)

          10
EMBOSS RGGGEKLFF
       : ::. : :
sp|P06 RDGGSSLGF
           1350
Waterman-Eggert score: 17;  11.8 bits; E(1) <  0.99
45.5% identity (63.6% similar) in 11 aa overlap (2-12:886-896)

              10
EMBOSS ASRDRGGGEKL
       .:\ : : :.:
sp|P06 VSYRRYGDEEL
         890

>>sp|P47871|GLR_HUMAN Glucagon receptor OS=Homo sapiens
          10
EMBOSS RGGGEK
       ::::..
sp|P47 RGGGSQ
```

A2: CASSAETQYF

capsid protein, partial [Coxsackievirus B4] APZ85768.1

```
Query  3   SSAET  7
           SSAET
Sbjct  38  SSAET  42
```

polyprotein, partial [Coxsackievirus B2] CCW33458.1

```
Query  2    ASSAETQY  9
            AS+A T+Y
Sbjct  986  ASTAKTEY  993
```

polyprotein, partial [Coxsackievirus B5] CCW33415.1

```
Query  2    ASSAETQY  9
            AS+A T+Y
Sbjct  987  ASTAKTEY  994
```

polyprotein [Coxsackievirus B3] AUF49642.1

```
Query  2     ASSAETQY  9
             AS+A T+Y
Sbjct  1555  ASTAKTEY  1562
```

type VII secretion protein EssB [Clostridium perfringens] : NWJ12916.1

```
Query  3    SSAETQY  9
            SSAETQY
Sbjct  248  SSAETQY  254
```

hypothetical protein [Clostridioides difficile] WP_077707125.1

23.5 bits(48) 816 6/6(100%) 6/6(100%) 0/6(0%)

```
Query  5   AETQYF  10
           AETQYF
Sbjct  27  AETQYF  32
```

```
>>sp|P06213|INSR_HUMAN Insulin receptor OS=Homo
              10
EMBOSS ASSAETQYF
       .:  .: \::
sp|P06 GSWTEPTYF
           940
```

```
>>sp|P47871|GLR_HUMAN Glucagon receptor OS=Homo

EMBOSS SSAET
       :::::
sp|P47 SSAET
      460
```

A3: CASSFRQGEQETQYF

polyprotein, partial [Coxsackievirus B5] UEU84385.1

```
Query  7     QGEQE-----TQYF  15
             QG+QE     +QYF
Sbjct  1170  QGDQEQLFSNVQYF  1183

Query  2    ASSFRQG  8
            AS F+QG
Sbjct  183  ASKFHQG  189

Query  10   QETQYF  15
            QE++Y+
Sbjct  936  QESEYY  941
```

polyprotein, partial [Coxsackievirus B2] : UEU84434.1

```
Query  7     QGEQE-----TQYF  15
             QG+QE     +QYF
Sbjct  1171  QGDQEQLFSNVQYF  1184

Query  2    ASSFRQG  8
            AS F+QG
Sbjct  183  ASKFHQG  189
```

polyprotein, partial [Coxsackievirus B4] QRG33154.1

```
Query  7     QGEQE-----TQYF  15
             QG+QE     +QYF
Sbjct  1168  QGDQEQLFSNVQYF  1181

Query  2    ASSFRQG  8
            AS F+QG
Sbjct  183  ASKFHQG  189

>>sp|P01308|INS_HUMAN Insulin OS=Homo sapiens OX=9606 GN  (110 aa)
>>sp|P01308|INS_HUMAN Insulin OS=Homo sapiens OX=9606 GN  (110 aa)
              10
EMBOSS ASSFRQGEQE
       :.... :. :
sp|P01 AEDLQVGQVE
        60

>>sp|P06213|INSR_HUMAN Insulin receptor OS=Homo sapiens   (1382 aa)

EMBOSS CASSFRQG
       : .: :::
sp|P06 CKNSRRQG
            300

Waterman-Eggert score: 24;  15.6 bits; E(1) <  0.33
57.1% identity (85.7% similar) in 7 aa overlap (7-13:1047-1053)

         10
EMBOSS QGEQETQ
       .:: ::.
sp|P06 KGEAETR
```

```
       1050

Waterman-Eggert score: 23;  15.1 bits; E(1) <  0.45
80.0% identity (100.0% similar) in 5 aa overlap (3-7:726-730)

EMBOSS SSFRQ
       ::::.
sp|P06 SSFRK
         730

>>sp|P01275|GLUC_HUMAN Pro-glucagon OS=Homo sapiens OX=9  (180 aa)
              10
EMBOSS ASSFRQGEQETQY
       .::. .:.   ..
sp|P01 VSSYLEGQAAKEF
        110

>>sp|Q99259|DCE1_HUMAN Glutamate decarboxylase 1 OS=Homo  (594 aa)
          10
EMBOSS RQGEQETQYF
       ..::..: \:
Sp|Q99 KNGEEQTVQF
        110
```

A4: CASSISNTGELFF

VP1, partial [Coxsackievirus B1] AEI98579.1

```
Query  1   CASSISNT  8
           CA+ ++NT
Sbjct  48  CATYTNNT  55

>>sp|Q16849|PTPRN_HUMAN Receptor-type tyrosine-protein p  (979 aa)
             10
EMBOSS SSISNTGELF
       :..:  : ::
sp|Q16 SAVSAHGCLF
             40
```

T-cell receptor beta V20-3J2.2, TCRBV20-3J2.2 {complementarity determining region 3} [human, peripheral T-lymphocytes, insulin-dependent diabetes mellitus patient GAD65.05,

```
Query  1   CASSIS--NTGELFF  13
           CA SI \ NTGELFF
Sbjct  1   CAWSIGSRNTGELFF  15
```

T-cell receptor beta V21-2J2.7, TCRBV21-2J2.7 {complementarity determining region 3} [human, peripheral T-lymphocytes, insulin-dependent diabetes mellitus patient GAD65.05,

```
Query  1  CASSISNTGE  10
```

```
          CASS S++GE
Sbjct  1  CASSQSHSGE  10
```

A5: CASSLLAGDNEQFF

polyprotein, partial [Coxsackievirus B5] UEU84385.1

```
Query  8     GDNEQFF  14
             GD+EQ F
Sbjct  1171  GDQEQLF  1177
```

polyprotein, partial [Coxsackievirus B4] QRG33154.1
Sequence ID: Length: 1210Number of Matches: 1

```
Query  8     GDNEQFF  14
             GD+EQ F
Sbjct  1169  GDQEQLF  1175
```

polyprotein, partial [Coxsackievirus B2] UEU84434.1
Sequence ID: Length: 1221Number of Matches: 1

```
Query  8     GDNEQFF  14
             GD+EQ F
Sbjct  1172  GDQEQLF  1178
```

polyprotein [Coxsackievirus B1] AKF40402.1
Sequence ID: Length: 2182Number of Matches: 1

```
Query  8     GDNEQFF  14
             GD+EQ F
Sbjct  1168  GDQEQLF  1174
```

efflux RND transporter periplasmic adaptor subunit [Clostridium perfringens] WP_221372394.1

```
Query  6    LAGDNEQF  13
            L GDNE+F
Sbjct  310  LSGDNEEF  317
```

tetanus toxoid-specific T-cell receptor beta chain [human, peripheral blood mononuclear cells, clone AKG6, Peptide Partial, 115 aa] [Homo sapiens] AAB25559.1

```
Query  1    CASSL-LAGDNEQFF  14
            CASSL LAG NEQFF
Sbjct  91   CASSLKLAGGNEQFF  105
```

```
>>sp|P47871|GLR_HUMAN Glucagon receptor OS=Homo sapiens
EMBOSS ASSLLAGD
       :::.:. :
sp|P47 ASSVLVID
       190
```

```
EMBOSS ASSLLAG
       :.. :::
sp|P47 AETPLAG
```

myosin IA [Homo sapiens] KAI2566481.1

```
Query  5   LLAGDNEQ  12
           LLAG +EQ
Sbjct  42  LLAGADEQ  49
```

A6: CASSLPGGAGNEQFF

capsid protein VP1, partial [Coxsackievirus B6] AEX14714.1

```
Query  2   ASSLPGGAGN  11
           A++LPGG  N
Sbjct  15  ADTLPGGPSN  24
```

hemolysin A [Clostridioides difficile] VTQ90367.1

```
Query  2    ASSLPGGAGNEQFF  15
            AS LPG  GN +FF
Sbjct  230  ASPLPGPSGNVEFF  243
```

helix-turn-helix transcriptional regulator [Clostridioides difficile] HBF9370847.1

```
Query  4   SLPGGAGNEQ  13
           S+ GGAGNE+
Sbjct  63  SIAGGAGNEE  72
```

Lysozyme family protein [Clostridioides difficile] HBH3061466.1

```
Query  1    CASSLPGGAGN  11
            CA SL GG+GN
Sbjct  270  CAVSLFGGGGN  280
```

```
>>sp|Q99259|DCE1_HUMAN Glutamate decarboxylase 1 OS=Homo
           10
EMBOSS LPGGAGNEQ
       ::.  :.::
sp|Q99 LPAKNGEEQ
           110
```

```
>>sp|P01275|GLUC_HUMAN Pro-glucagon OS=Homo sapiens
               10
EMBOSS ASSLPGGAGNE
       .: : : :..:
sp|P01 SSYLEGQAAKE
       110
```

```
>>sp|P06213|INSR_HUMAN Insulin receptor OS=Homo sapiens    (
              10
EMBOSS ASSLPGGAG
       :. : :.::
sp|P06 AALLLGAAG
       20
```

T-cell receptor beta V21-1J2.5, TCRBV21-1J2.5 {complementarity determining region 3} [human, peripheral T-lymphocytes, insulin-dependent diabetes mellitus patient GAD65.05,

```
Query  1    CASSLPGGAGNE-QFF  15
            CASS PG  G+E Q+F
Sbjct  1    CASSWPG--GHETQYF  14
```

T-cell receptor beta V6-2J1.4, TCRBV6-2J1.4 {complementarity determining region 3} [human, peripheral T-lymphocytes, insulin-dependent diabetes mellitus patient GAD65.05, Peptide Query 1 CASSLPGGA-GNEQFF 15

```
            CASS PG A G+E FF
Sbjct  1    CASSPPGRADGDELFF  16
```

A7: CASSPPGLGEQYF

polyprotein, partial [Coxsackievirus B5] CCW33443.1

```
Query  1     CASSP  5
             CASSP
Sbjct  508   CASSP  512
```

polyprotein [Coxsackievirus B4] WHA31326.1

```
Query  1      CASSP  5
              CASSP
Sbjct  1074   CASSP  1078

Query  3     SSPPGL  8
             SSP GL
Sbjct  795   SSPGGL  800
```

polyprotein [Coxsackievirus B3] SPS68031.1

```
Query  1      CASSP  5
              CASSP
Sbjct  1076   CASSP  1080
```

polyprotein [Coxsackievirus B2] BBA80352.1

```
Query  1      CASSP  5
              CASSP
Sbjct  1078   CASSP  1082
```

polyprotein [Coxsackievirus A9] AUF49631.1
```
Query  1     CASSP  5
             CASSP
Sbjct  1092  CASSP  1096
```

polyprotein [Coxsackievirus A6] WAW78347.1
```
Query  2    ASSPP  6
            ASSPP
Sbjct  203  ASSPP  207

Query  3    SSPPG--LGEQY  12
            S  PG  L  QY
Sbjct  704  STTPGMLL--QY  713
```

VP1 polyprotein, partial [Coxsackievirus B2] UWV17742.1
```
Query  2   ASSPPG  7
           ASS PG
Sbjct  78  ASSTPG  83
```

hypothetical protein [Clostridioides difficile] HBN6128498.1
```
Query  2   ASSPPGLG  9
           AS+PPGLG
Sbjct  54  ASTPPGLG  61
```

macro domain-containing protein [Clostridium perfringens] WP_143860178.1
```
Query  8    LGEQYF  13
            LGEQYF
Sbjct  193  LGEQYF  198
```

collagenase [Clostridium botulinum C str. Stockholm] EGO88204.1
```
Query  8    LGEQYF  13
            LGEQYF
Sbjct  297  LGEQYF  302
```

```
>>sp|P47871|GLR_HUMAN Glucagon receptor OS=Homo sapiens   (

EMBOSS ASSPPGLG
       ::: :: :
sp|P47 ASSSPGHG
          440
```

tropomyosin 1 (alpha), isoform CRA_l [Homo sapiens] EAW77632.1
```
Query  2   ASSPP-GLGE  10
           A SPP GLGE
Sbjct  60  AGSPPRGLGE  69
```

T-cell receptor beta V6-2J1.4, TCRBV6-2J1.4 {complementarity determining region 3} [human, peripheral T-lymphocytes, insulin-dependent diabetes mellitus patient GAD65.05, AAB46915.1
```
Query  1  CASSPPG  7
          CASSPPG
Sbjct  1  CASSPPG  7
```

A8: CASSPRDGSSYNEQFF

polyprotein, partial [Coxsackievirus A9] BAE44079.1
```
Query  1   CASSP  5
           CASSP
Sbjct  65  CASSP  69
```

VP1, partial [Coxsackievirus A10] UYR00073.1
```
Query  2    ASSPRDGSS  10
            A S RD SS
Sbjct  284  ANSARDSSS  292
```

polyprotein, partial [Coxsackievirus B5] CCW33443.1
```
Query  1    CASSP  5
            CASSP
Sbjct  508  CASSP  512
```

polyprotein [Coxsackievirus B4] ANY27029.1
```
Query  1     CASSP  5
             CASSP
Sbjct  1074  CASSP  1078
```

polyprotein [Coxsackievirus B3] SPS68031.1
```
Query  1     CASSP  5
             CASSP
Sbjct  1076  CASSP  1080
```

polyprotein [Coxsackievirus B2] BBA80352.1
```
Query  1     CASSP  5
             CASSP
Sbjct  1078  CASSP  1082
```

DUF4145 domain-containing protein [Clostridium perfringens] MDM0977544.1
```
Query  6    RDGSSYNE  13
            RDGS YNE
Sbjct  422  RDGSKYNE  429

Query  6    RDGSSYNE  13
            R+GS YNE
Sbjct  338  RNGSKYNE  345

Query  6    RDGSSYNE  13
            R+GS YNE
Sbjct  380  RNGSKYNE  387
```

reverse transcriptase domain-containing protein [Clostridium perfringens] WP_158524188.1
```
Query  9    SSYNEQF  15
            S YNEQF
Sbjct  203  SNYNEQF  209
```

DNA topoisomerase III [Clostridioides difficile] HAU5417622.1
```
Query  7   DGSSYNEQF  15
           D   YNEQF
Sbjct  48  DAATYNEQF  56
```

```
Query  9    SSYNEQF  15
            S YNEQF
Sbjct  218  SNYNEQF  224
```

type I restriction modification DNA specificity domain protein [Clostridioides difficile P28] EQJ55303.1

```
Query  4   SPRDGSSYNEQ-----FF  16
           SP  GSSYNE+     FF
Sbjct  26  SPS-GSSYNEESIGTIFF  42
```

coenzyme F420-0:L-glutamate ligase [Clostridioides difficile] WP_271641823.1

```
Query  2    ASSPRDGSSYNEQF  15
            ASS  DGS YN+Q+
Sbjct  225  ASSV-DGSGYNDQY  237

>>sp|P06213|INSR_HUMAN Insulin receptor OS=Homo sapiens

EMBOSS CASSPRDGS
       : :.:. \:
sp|P06 CCSCPKTDS
      710

              10
EMBOSS ASSPRDGSSYNEQ
       :..: .: : :::
sp|P06 AEQPYQGLS-NEQ
           1240


>>sp|P01275|GLUC_HUMAN Pro-glucagon OS=Homo sapiens OX=9  (
         10
EMBOSS DGSSYNE
       : ::: :
sp|P01 DVSSYLE
         110

         10
EMBOSS DGSSYNE
       :::/\.:
sp|P01 DGSFSDE
       150

              10
EMBOSS ASSPRDGSSYNE
       .::  .:.. .:
sp|P01 VSSYLEGQAAKE
        110


>>sp|P47871|GLR_HUMAN Glucagon receptor OS=Homo sapiens   (
              10
EMBOSS ASSPRDGSSYNE
       .:::  :  \.:
sp|P47 SSSPGHGPPSKE
         440
```

```
>>sp|Q16849|PTPRN_HUMAN Receptor-type tyrosine-protein p
          10
EMBOSS RDGSSYNE
       ::::  .:
sp|Q16 RDGSRVSE
        220
```

A9: CASSYGTVNSPLHF

3D protein, partial [Coxsackievirus A6] QHJ89586.1

```
Query  5    YGTVN  9
            YGTVN
Sbjct  102  YGTVN  106
```

polyprotein [Coxsackievirus A7] ADZ11086.1

```
Query  5     YGTVN  9
             YGTVN
Sbjct  1832  YGTVN  1836
```

polyprotein [Coxsackievirus A10] USC32123.1
Sequence ID: Length: 2193Number of Matches: 1

```
Query  5     YGTVN  9
             YGTVN
Sbjct  1833  YGTVN  1837
```

polyprotein, partial [Coxsackievirus A1] WBR19144.1

```
Query  6    GTVNS  10
            GTVNS
Sbjct  399  GTVNS  403
```

polyprotein, partial [Coxsackievirus B2] BAD11195.1

```
Query  5   YGTVNSPLHF  14
           YG  NS LH+
Sbjct  20  YG--NSLLHY  27
```

hypothetical protein [Clostridium perfringens] MDK0841415.1

```
Query  5   YGTVNSPL  12
           YGT+N+PL
Sbjct  54  YGTINTPL  61
```

diguanylate kinase [Clostridioides difficile] KPI54855.1

```
Query  3    SSYGTVNSPLH  13
            SSYGTVN+ L+
Sbjct  120  SSYGTVNTVLQ  130
```

transporter substrate-binding domain-containing protein [Clostridioides difficile] MDI0267395.1

```
Query  3    SSYGTVNSPLH  13
            SSYGTVN+ L+
Sbjct  120  SSYGTVNTVLQ  130
```

hemolysin III-like protein [Clostridioides difficile] VTR07327.1

```
Query  2   ASSYGTVNS  10
           ASSYGTV S
Sbjct  12  ASSYGTVGS  20
```

replication endonuclease, partial [Clostridioides difficile] WP_032509971.1

```
Query  1    CASSYGTVNSPLHF  14
            C+ +YGT+N  LHF
Sbjct  120  CVPEYGTANGRLHF  133
```

```
>>sp|P06213|INSR_HUMAN Insulin receptor OS=Homo sapiens
               10
EMBOSS CASSYGTVNS
       : : : :.::
sp|P06 CPSGY-TMNS
           320

          10
EMBOSS GTVNSPLHF
       :.  ..: .:
sp|P06 GSWTEPTYF
           940

>>sp|P47871|GLR_HUMAN Glucagon receptor OS=Homo sapiens
              10
EMBOSS ASSYGTVNSPLH
       .:.. . .:: :
sp|P47 TSNHRASSSPGH
     430       440
```

A10: CATGLAANTGELFF

polyprotein [Coxsackievirus B5] AVJ35742.1

```
Query  3   TGLAAN  8
           TGL+AN
Sbjct  15  TGLSAN  20

Query  6    AANTG  10
            AA+TG
Sbjct  601  AAETG  605
```

```
Query  5     LAANT  9
             LA+NT
Sbjct  1642  LAINT  1646
```

VP1, partial [Coxsackievirus A22] AFN66229.1

```
Query  6   AANTGELF  13
           A NT++LF
Sbjct  38  ADNTADLF  45
```

VP1, partial [Coxsackievirus A10] QNN25656.1

```
Query  5   LAANT  9
           LAANT
Sbjct  28  LAANT  32

Query  6   AANTG  10
           AA+TG
Sbjct  49  AAETG  53
```

DUF4956 domain-containing protein, partial [Clostridium perfringens] WP_265881915.1

```
Query  3    TGLAANTGELF  13
            TGL A+TGE+F
Sbjct  114  TGLSASTGEIF  124
```

collagen-like protein [Clostridioides difficile] NJI82831.1

```
Query  2   ATGLAANTGE  11
           ATGL++NTGE
Sbjct  49  ATGLTGNTGE  58
```

```
>sp|P06213|INSR_HUMAN Insulin receptor OS=Homo sapiens
           10
EMBOSS LAANTGELFF
       :. . :.:::
sp|P06 LTITQGKLFF
         450

EMBOSS ATGLAAN
       :: ::.:
sp|P06 ATSLAGN
        930
              10
EMBOSS CATGLAANTGEL
       : .: . :.  :
sp|P06 CPSGYTMNSSNL
          320
             10
EMBOSS ATGLAANTG
       :. : :: :
sp|P06 AAELEANLG
       380
```

A11: CSVNRGGTDTQYF

polyprotein [Coxsackievirus A10] AXF74005.1

```
Query  6    GGTD-TQY  12
            GGTD T+Y
Sbjct  662  GGTDTTEY  669
```

VP1, partial [Coxsackievirus A4] AEP43815.1

```
Query  1   CSVNRGGT-DT--QYF  13
           C VN  GT +T  +YF
Sbjct  1   CVVNSNGTRETHVEYF  16
```

peptide-methionine (S)-S-oxide reductase [Clostridioides difficile P28] EQJ59459.1

```
Query  2    SVNRGGTDT--QY  12
            SVNR G+DT  QY
Sbjct  120  SVNRQGNDTGSQY  132
```

glycoside hydrolase family 31 protein, partial [Clostridium perfringens] WP_274541760.1

```
Query  7   GTDTQY  12
           GTDTQY
Sbjct  60  GTDTQY  65
```

```
>>sp|Q16849|PTPRN_HUMAN Receptor-type tyrosine-protein p
               10
EMBOSS CSVNRGGTDT
       ::   : : :
sp|Q16 CSDGAGRTGT
      910
```

TCR DIA 1 CSVEATRADTQYFG

polyprotein [Coxsackievirus B5] QQL13737.1

Sequence ID: Length: 2185Number of Matches: 4

```
Query  7     RADTQY  12
             RAD QY
Sbjct  2103  RADDQY  2108
```

polyprotein, partial [Coxsackievirus B3] UPI41528.1

```
Query  7     RADTQY  12
             RAD QY
Sbjct  2103  RADDQY  2108
```

30S ribosomal protein S2 [Clostridium perfringens] NWJ13795.1

```
Query  4   EATRADTQYF  13
           EATRA  QYF
Sbjct  83  EATRAG-QYF  91
```

M4 family metallopeptidase [Clostridium perfringens] WP_003463430.1

```
Query  7    RADTQYF  13
            RA TQYF
Sbjct  506  RATTQYF  512
```

lambda toxin [Clostridium perfringens] BAA08311.1

```
Query  7    RADTQYF  13
            RA+TQYF
Sbjct  506  RATTQYF  512
```

alpha-galactosidase [Clostridium perfringens] WP_279857802.1

```
Query  2    SVEATRADTQYFG  14
            S+E TRA++ YFG
Sbjct  589  SLE-TRANVAYFG  600

>>sp|P01275|GLUC_HUMAN Pro-glucagon OS=Homo sapiens OX=9

EMBOSS SVEATRAD
       : .:..::
sp|P01 SFSASQAD

>>sp|Q99259|DCE1_HUMAN Glutamate decarboxylase 1 OS=Homo  (
             10
EMBOSS VEATRADTQY
       : :: . : :
sp|Q99 VNATAGTTVY
              350


>>sp|Q16849|PTPRN_HUMAN Receptor-type tyrosine-protein p
              10
EMBOSS SVEATRADTQY
       ... ::. ::.
sp|Q16 QTQETRTLTQF
     860       870
             10
EMBOSS VEATRADTQ
       .:.: : :.
sp|Q16 AEGTPASTR
        880
            10
EMBOSS EATRADTQY
       :....:: .
sp|Q16 EGAHGDTTF
       620
               10
EMBOSS CSVEATRADT
       ::  : :. :
sp|Q16 CSDGAGRTGT
```

```
      910
```

T-cell receptor beta V21-1J2.5, TCRBV21-1J2.5 {complementarity determining region 3} [human, peripheral T-lymphocytes, insulin-dependent diabetes mellitus patient GAD65.05, Peptide Partial, 23 aa] [Homo sapiens] AAB46917.1

```
Query  9    DTQYFG  14
            +TQYFG
Sbjct  10   ETQYFG  15
```

TCR DIA 2   CASSLAVIRTDTQYFG

VP1, partial [Coxsackievirus A24] ADA84739.1

```
Query  12   TQYFG  16
            TQYFG
Sbjct  49   TQYFG  53
```

RNA-dependent RNA polymerase, partial [Coxsackievirus B4] AAR16059.1

```
Query  9     RTDTQY  14
             RTD+QY
Sbjct  141   RTDEQY  146
```

polyprotein, partial [Coxsackievirus A13] UBM82664.1

```
Query  3     SSLAVI  8
             SSLA+I
Sbjct  461   SSLAII  466
```

polyprotein, partial [Coxsackievirus B3] UPI41540.1

```
Query  9      RTDTQY  14
              RTD+QY
Sbjct  1533   RTDEQY  1538

Query  2      ASSL  5
              ASSL
Sbjct  1331   ASSL  1334
```

polyprotein [Coxsackievirus B5] QQL13741.1

```
Query  9      RTDTQY  14
              RTD+QY
Sbjct  2103   RTDEQY  2108

Query  2      ASSL  5
              ASSL
Sbjct  1901   ASSL  1904
```

polyprotein [Coxsackievirus B2] : BBA80346.2

```
Query  9     RTDTQY  14
             RTD QY
Sbjct  2105  RTDEQY  2110
```

site-2 protease family protein [Clostridium perfringens] WP_208364430.1

```
Query  2    ASSLAVI----RTDTQYFG  16
            A SL+VI    /\+T+YFG
Sbjct  112  AASLGVIGVLVSKNTEYFG  130
```

hypothetical protein [Clostridium perfringens] WP_075823906.1

```
Query  4    SLAVIRTDTQY  14
            SLAV+ T+T Y
Sbjct  258  SLAVVITETKY  268
```

NUDIX domain-containing protein [Clostridium perfringens] EGT3616113.1

Sequence ID: Length: 147Number of Matches: 1

```
Query  6   AVIRTDTQYF  15
           AV+RT+T YF
Sbjct  33  AVVRTSTGYF  42
```

minor capsid protein [Clostridium perfringens] WP_110082846.1

```
Query  4    SLAVIRTDT  12
            SL VIRT+T
Sbjct  180  SLRVIRTET  188
```

prolipoprotein diacylglyceryl transferase [Clostridium perfringens] ELC8365287.1

```
Query  8    IRTDTQYF  15
            IRTD+ YF
Sbjct  217  IRTDSLYF  224
```

hypothetical protein LZD60_16220 [Clostridium perfringens] WCM70067.1

```
Query  7    VIRTDT  12
            VIRTDT
Sbjct  217  VIRTDT  222
```

DNA mismatch repair protein [Clostridium botulinum] MBN1069351.1

```
Query  3    SSLAVIRTDTQY  14
            SSLA+IR D  Y
Sbjct  392  SSLAIIRYDNKY  403
```

phage N-6-adenine-methyltransferase [Clostridium botulinum] WP_012047611.1

Sequence ID: Length: 140Number of Matches: 1

```
Query  9    RTDTQYF  15
            RTDT YF
Sbjct  98   RTDTRYF  104
```

```
>>sp|P06213|INSR_HUMAN Insulin receptor OS=Homo sapiens   (
            10
EMBOSS SLAVIRTDTQYF
       .:.::: .  .:
sp|P06 NLTVIRGSRLFF
          110
            10
```

```
EMBOSS SLAVIRTDTQYFG
       .: .:: .:  .:
sp|P06 KLRLIRGETLEIG
              420

>>sp|P47871|GLR_HUMAN Glucagon receptor OS=Homo sapiens

EMBOSS ASSLAVI
       :::..::
sp|P47 ASSVLVI
       190
             10
EMBOSS SSLAVIRTDTQY
       ..:.  :: ..:
sp|P47 TELVCNRTFDKY
            60
```

T-cell receptor beta V21-1J2.5, TCRBV21-1J2.5 {complementarity determining region 3} [human, peripheral T-lymphocytes, insulin-dependent diabetes mellitus patient GAD65.05, Peptide Partial, 23 aa] [Homo sapiens] AAB46917.1

```
Query  11  DTQYFG  16
           +TQYFG
Sbjct  10  ETQYFG  15
```

TCR DIA 3   CAVGALAGTASKLTG

polyprotein [Coxsackievirus A6] QKZ25788.1
Sequence ID: Length: 2201Number of Matches: 2

```
Query  2     AVGALAGT  9
             AV ALAGT
Sbjct  576   AVSALAGT  583

Query  7      AGTASKL  13
              AG A KL
Sbjct  1382  AGRAAKL  1388
```

pyruvate oxidase [Clostridium perfringens] : NWJ14174.1

```
Query  3   VGALAGTA-SKLTG  15
           VGALA   A  KLTG
Sbjct  54  VGALAAAADAKLTG  67
```

MFS transporter [Clostridium perfringens] WP_283694243.1

```
Query  3    VGALAGTASK  12
            VGAL GT SK
Sbjct  312  VGALSGTVSK  321
```

dihydroorotate dehydrogenase [Clostridium perfringens] MDO4534443.1
```
Query  3   VGALAGTASK-LT  14
           VGAL+G+ SK LT
Sbjct  34  VGALGGISSKGLT  46
```

DNA polymerase III subunit alpha [Clostridium perfringens] EJT5937192.1
```
Query  3     VGALAGTASKLT  14
             +G +AGT +KLT
Sbjct  1075  IGSIAGTNTKLT  1086
```

Blp family class II bacteriocin [Clostridium perfringens] WP_139934343.1
```
Query  2   AVGALAGT  9
           AVGAL+GT
Sbjct  50  AVGALVGT  57
```

SH3 domain-containing protein, partial [Clostridium perfringens] WP_270204657.1
```
Query  7    AGTASKLTG  15
            AGT SK+TG
Sbjct  316  AGTSSKVTG  324
```

tape measure protein [Clostridium perfringens] EJT5929836.1
```
Query  3    VGALAG----TASKL  13
            VGALAG    +A KL
Sbjct  879  VGALAGGILLVAAKL  893
```

DUF421 domain-containing protein [Clostridium botulinum] WP_012344633.1
```
Query  3   VGALAGTAS  11
           +GA+AGTAS
Sbjct  44  IGAIAGTAS  52
```

hydroxyethylthiazole kinase [Clostridium botulinum] WP_043989494.1
```
Query  1    CAVGAL-AGTAS  11
            C VGAL A+TAS
Sbjct  175  CMVGALTAATAS  186
```

```
>>sp|P06213|INSR_HUMAN Insulin receptor OS=Homo sapiens
           10
EMBOSS ALAGTAS
       .:::..:
sp|P06 SLAGNGS
      930

EMBOSS CAVGA
       :.:.:
sp|P06 CSVAA
               10
EMBOSS AVGALA-GTASKL
       ::.::  :.:..:
sp|P06 AVAALLLGAAGHL
         20
          10
EMBOSS LAGTASK
       : :..::
sp|P06 LLGVVSK
     1090
```

```
              10
EMBOSS AVGALAGTAS
       :.:.  :.:.
sp|P06 ATGGRRGAAA
              10
              10
EMBOSS AVGALAGTASKLTG
       :.  :...:. : :
sp|P06 AAPLLVAVAALLLG
               20
              10
EMBOSS AVGALAGTAS
       .:.:. .:.:
sp|P06 TVAAFPNTSS
         780


>>sp|P47871|GLR_HUMAN Glucagon receptor OS=Homo sapiens
           10
EMBOSS ALAGTASKL
       :..: .:::
sp|P47 AILGGLSKL


>>sp|Q99259|DCE1_HUMAN Glutamate decarboxylase 1 OS=Homo

EMBOSS VGALAGT
       :.: :::
sp|Q99 VNATAGT

>>sp|Q16849|PTPRN_HUMAN Receptor-type tyrosine-protein p
           10
EMBOSS ALAGTASKL
       ::::.:. :
sp|Q16 ALAGVAGLL
            590
         10
EMBOSS AGTASKLT
       ::..:.:.
sp|Q16 AGASSSLS
        170
```

zinc transporter SLC39A7 isoform 2 [Homo sapiens] NP_001275706.1

```
Query  2    AVGALAGTA  10
            AVGALAGTA
Sbjct  263  AVGALAGTA  271
```

KE4 protein [Homo sapiens] AAD12305.1

```
Query  2    AVGALAGTA  10
            AVGALAGTA
Sbjct  388  AVGALAGTA  396
```

myosin, heavy polypeptide 7B, cardiac muscle, beta, isoform CRA_c [Homo sapiens] EAW76238.1

```
Query  2    AVGALA  7
            AVGALA
```

```
Sbjct  418  AVGALA  423
```

intermembrane lipid transfer protein VPS13A isoform A [Homo sapiens] NP_150648.2

```
Query  2     AVGALAGTASKLTG  15
             AVG+LAG+ASK+TG
Sbjct  2913  AVGGLAGAASKITG  2926
```

vacuolar protein sorting 13 homolog A [Homo sapiens] KAI2552925.1

```
Query  2     AVGALAGTASKLTG  15
             AVG LAG ASK TG
Sbjct  2913  AVGGLAGAASKITG  2926
```

chorea-acanthocytosis [Homo sapiens] AAK61861.1

```
Query  2     AVGALAGTASKLTG  15
             AVG LAG ASK TG
Sbjct  2913  AVGGLAGAASKITG  2926
```

TCR diabetic 4: CASSLATSGGGSDTQYFG

Chain E, The light chain of fab 9B5 [Coxsackievirus A16] 7YMS_E

```
Query  8   SGGGSDTQY  16
           SG GS TQY
Sbjct  63  SGSGSGTQY  71

Query  7    TSGGGS  12
            TSGG+S
Sbjct  126  TSGGAS  131
```

polyprotein, partial [Coxsackievirus B3] ADW53828.1

```
Query  8    SGGGSDTQYFG  18
            S GGS TQ FG
Sbjct  406  SNGGSGTQVFG  416
```

polyprotein, partial [Coxsackievirus B3] QRG33196.1

```
Query  14    TQYFG  18
             TQY+G
Sbjct  1089  TQYYG  1093

Query  11   GSDTQYFG  18
            G+ TQ FG
Sbjct  409  GTGTQVFG  416
```

polyprotein [Coxsackievirus B5] AKD28015.1

```
Query  4    SLATSGGGSDTQYFG  18
            S+AT+G    TQ FG
Sbjct  404  SIATNG----TQVFG  414
```

```
Query  5     LATSGGGS  12
             LA++ +GS
Sbjct  1319  LASTNAGS  1326
```

M20/M25/M40 family metallo-hydrolase, partial [Clostridium perfringens] WP_238001092.1

```
Query  8   SGGGSDT  14
           SGGGSDT
Sbjct  92  SGGGSDT  98
```

peptidase M20 [Clostridium perfringens] PWX11336.1

```
Query  8    SGGGSDT  14
            SGGGSDT
Sbjct  303  SGGGSDT  309
```

beta-galactosidase [Clostridium perfringens] CAG9352954.1

```
Query  11  GSDT-QYF  17
           GSDT QYF
Sbjct  33  GSDTVQYF  40
```

phage/plasmid primase, P4 family [Clostridium perfringens] WP_197926775.1

```
Query  10   GGS-DTQYF  17
            GGS +TQYF
Sbjct  244  GGSLNTQYF  252
```

M20/M25/M40 family metallo-hydrolase [Clostridium tetani] WP_035111056.1

```
Query  6    ATSGGGSD  13
            A SGGGSD
Sbjct  315  AASGGGSD  322
```

phage tail tape measure protein [Clostridium botulinum] WP_205427851.1

```
Query  2    ASSLATSG  9
            ASSLAT G
Sbjct  563  ASSLATAG  570

Query  2     ASSLATSGGGS  12
             A S+A /GGGS
Sbjct  1406  APSIASGGGGS  1416

>>sp|P06213|INSR_HUMAN Insulin receptor OS=Homo sapiens   (
              10
EMBOSS ASSLATSGGGSDTQYF
       :.::: .:. .. .::
sp|P06 ATSLAGNGSWTEPTYF
        930       940
         10
EMBOSS TSGGGSDTQY
       : :. ::. :
sp|P06 TYGAKSDIIY
          610
            10
EMBOSS SLATSGGGSDTQ
       . . .:.:: :.
sp|P06 ATSLAGNGSWTE
        930
```

```
           10
EMBOSS ATSGGGSD
       ..:: :..
sp|P06 TSSGTGAE
          750

>>sp|P47871|GLR_HUMAN Glucagon receptor OS=Homo sapiens   (
               10
EMBOSS ASSLATSGGGSDTQYFG
       :::    :  :  . \::
sp|P47 ASSSPGHGPPSKELQFG
          440       450
             10
EMBOSS SLATSGGGSDT
       ...  .::..:.
sp|P47 QFGRGGGSQDS
      450
             10
EMBOSS SLATSGGGS
       .::. :: :
sp|P47 ALAILGGLS
      160


>>sp|Q99259|DCE1_HUMAN Glutamate decarboxylase 1 OS=Homo
               10
EMBOSS ASSLATSGGGSD
       .:: ..:..:.:
sp|Q99 SSSATSSNAGAD
         10
               10
EMBOSS ASSLATSGGGS
       :::  .:.. :
sp|Q99 ASSTPSSSATS
               10


>>sp|Q16849|PTPRN_HUMAN Receptor-type tyrosine-protein p  (
              10
EMBOSS SSLATSGGGSDTQY
       ..:. .:. .:: .
sp|Q16 AALGPEGAHGDTTF
             620
              10
EMBOSS SSLATSGG
       ..:..:::
sp|Q16 GGLGGSGG
        10
```

Chain E, T-CELL-RECEPTOR, A3.10-beta chain [Homo sapiens] [6XC9_E (Tran et al., 2021)]

```
Query  1    CASSLATSGGGSDTQYFG  18
            CASSL  SGG  DTQYFG
Sbjct  90   CASSLSASGGATDTQYFG  107
```

Tran,M.T., Faridi,P., Lim,J.J., Ting,Y.T., Onwukwe,G.,
Bhattacharjee,P., Jones,C.M., Tresoldi,E., Cameron,F.J., La

```
            Gruta,N.L., Purcell,A.W., Mannering,S.I., Rossjohn,J. and
Reid,H.H.
  TITLE     T cell receptor recognition of hybrid insulin peptides bound to
            HLA-DQ8
  JOURNAL   Nat Commun 12 (1), 5110 (2021)
```

TCR diabetic 5: CAVVNMDSNYQ

Chain A, 2C protein [Coxsackievirus B3] 6S3A_A

```
Query  2    AVVNMD  7
            AVV MD
Sbjct  57   AVVIMD  62
```

penicillin-binding transpeptidase domain-containing protein [Clostridium perfringens] WP_224374358.1

```
Query  5     NMDSNYQ  11
             N+DSNYQ
Sbjct  724   NLDSNYQ  730
```

DNA topoisomerase IV subunit A [Clostridium tetani] WP_035140929.1

```
Query  3     VVNMDSNY  10
             VVNM+ NY
Sbjct  148   VVNMEDNY  155
```

AAA family ATPase [Clostridium perfringens] WP_198630792.1

```
Query  4     VNMDSNY  10
             VNMD NY
Sbjct  594   VNMDENY  600
```

flavodoxin-dependent (E)-4-hydroxy-3-methylbut-2-enyl-diphosphate synthase [Clostridium perfringens] MDM0468235.1

```
Query  1    CAVVNMDSNY  10
            CAV++MD  Y
Sbjct  55   CAVLDMDAAY  64
```

pyridoxal-dependent decarboxylase [Clostridium perfringens] WP_164812910.1

```
Query  2     AVVNMDSN  9
             A+VN+DSN
Sbjct  101   AIVNLDSN  108
```

CehA/McbA family metallohydrolase [Clostridium perfringens] EJT6500362.1

```
Query  3      VVNM-DSN  9
              VVNM DSN
Sbjct  1445   VVNMDDSN  1452
```

carbohydrate kinase family protein [Clostridium botulinum] EJO5346805.1

```
Query  2    AVVNMDS  8
            AVV+MDS
Sbjct  114  AVVDMDS  120
```

```
>>sp|P47871|GLR_HUMAN Glucagon receptor OS=Homo sapiens
             10
EMBOSS VVNMDSNYQ
       :..: :..:
sp|P47 VAKMYSSFQ
           140
```

TCR diabetic 6: CADAGGTSYKLFG

polyprotein [Coxsackievirus A6] QKZ25766.1

```
Query  9     YKLFG  13
             YKLFG
Sbjct  1527  YKLFG  1531
```

VP1, partial [Coxsackievirus A20] AFV68031.1

```
Query  4   AGGTSYKLF  12
           AGGT \KLF
Sbjct  49  AGGTKSKLF  57
```

L-ribulose-5-phosphate 3-epimerase [Clostridium perfringens] NWJ14549.1

```
Query  1   CADAGGT  7
           CADAGGT
Sbjct  65  CADAGGT  71
```

TetR/AcrR family transcriptional regulator [Clostridium perfringens] EGT3616816.1
Sequence ID: Length: 189Number of Matches: 1

```
Query  7   TSYKLF  12
           TSYKLF
Sbjct  14  TSYKLF  19
```

phage tail family protein [Clostridium botulinum] NFI38802.1

```
Query  7   TSYKLF  12
           TSYKLF
Sbjct  20  TSYKLF  25
```

FtsW/RodA/SpoVE family cell cycle protein [Clostridium botulinum] EJP6472118.1

```
Query  4    AGGT-SYKLF  12
            AG T SYKLF
Sbjct  235  AGATISYKLF  244
```

D-alanyl-D-alanine carboxypeptidase [Clostridium perfringens] MDO4534228.1
Sequence ID: Length: 460Number of Matches: 1

```
Query  8    SYKLFG  13
            SYKLFG
Sbjct  336  SYKLFG  341
```

glycoside hydrolase family 16 protein [Clostridium perfringens] MDM0455782.1

```
Query  5    GGTSYK  10
            GGTSYK
Sbjct  368  GGTSYK  373
```

```
>>sp|P47871|GLR_HUMAN Glucagon receptor OS=Homo sapiens   (
              10
EMBOSS AGGT--SYKLF
       : ::  : :::
sp|P47 AQGTLRSAKLF
            380


>>sp|Q99259|DCE1_HUMAN Glutamate decarboxylase 1 OS=Homo
               10
EMBOSS CADAGGTSYKL
       :. : : . ::
sp|Q99 CGVAHGCTRKL
             40
              10
EMBOSS ADAGGTSYKLF
       : :: : :  :
sp|Q99 ATAGTTVYGAF
            350
```

TCR diabetic 7: CIPGSEEYGNKLVFG

MAG: polyprotein, partial [Coxsackievirus A19] AWU65853.1

```
Query  7     EYGNKL--VFG  15
             +Y NKL  +FG
Sbjct  1031  DYVNKLGMAFG  1041
```

hypothetical protein [Clostridium perfringens] HAT4365449.1

```
Query  6    EEY--GNKLVFG  15
            E Y  GNKLVFG
Sbjct  739  EAYWQGNKLVFG  750
```

exo-alpha-sialidase NanH [Clostridium perfringens] MDM0471704.1
```
Query  1    CIPGSEEYGNKL  12
            CIP   EYGNKL
Sbjct  333  CIP-YPEYGNKL  343
```

potassium-transporting ATPase subunit KdpC [Clostridium perfringens] WP_096515864.1
```
Query  3    PGSEEYGNKLV  13
            P+SEEY +KLV
Sbjct  95   PASEEY-EKLV  104
```

ferrous iron transport protein B [Clostridium tetani] WP_130006490.1
```
Query  7    EYGNKLVF  14
            EYGNK+ F
Sbjct  555  EYGNKMMF  562
```

5'-nucleotidase C-terminal domain-containing protein [Clostridium tetani] WP_023438975.1
```
Query  6    EEYGNK  11
            EEYGNK
Sbjct  226  EEYGNK  231
```

Chain A, Receptor-type tyrosine-protein phosphatase gamma [Homo sapiens] 2PBN_A
```
Query  5    SEEYGN  10
            SEEYGN
Sbjct  153  SEEYGN  158
```

anti sigma factor C-terminal domain-containing protein [Clostridium botulinum] HBJ1646806.1
```
Query  5    SEEYGNKLVF  14
            SEE+  KLVF
Sbjct  227  SEEFSSKLVF  236
```

```
>>sp|Q16849|PTPRN_HUMAN Receptor-type tyrosine-protein p

EMBOSS PGSEEYG
       :..::::
sp|Q16 PAAEEYG
         470
```

protein-tyrosine phosphatase, partial [Homo sapiens] CAA38070.1
```
Query  4    GSEEYGN  10
            GSEEYGN
Sbjct  366  GSEEYGN  372
```

Crystal structure of Human PTPRZ D1 domain [Homo sapiens] 5AWX_A
```
Query  4    GSEEYGN  10
            GSEEYGN
Sbjct  143  GSEEYGN  149
```

protein tyrosine phosphatase receptor type Z1, partial [Homo sapiens] KAI2547656.1
```
Query  4    GSEEYGN  10
            GSEEYGN
Sbjct  285  GSEEYGN  291
```

Insulin activator factor, partial [Homo sapiens] AAB31986.1

```
Query  6    EEYGNKL  12
            EEYG KL
Sbjct  568  EEYG-KL  573
```

human diabetogenic TCR T1D3 in complex with DQ8-p8E9E peptide [Homo sapiens] 6DFX_G (Wang, et al., 2019)

```
Query  9    GNKLVFG  15
            G KL+FG
Sbjct  98   GGKLIFG  104
```


```
Wang,Y., Sosinowski,T., Novikov,A., Crawford,F., White,J., Jin,N.,
            Liu,Z., Zou,J., Neau,D., Davidson,H.W., Nakayama,M., Kwok,W.W.,
            Gapin,L., Marrack,P., Kappler,J.W. and Dai,S.
  TITLE     How C-terminal additions to insulin B-chain fragments create
            superagonists for T cells in mouse and human type 1 diabetes
  JOURNAL   Sci Immunol 4 (34) (2019)
   PUBMED   30952805
```


TCR diabetic 8: CASSQVRLAGGGEQFFG

polyprotein, partial [Coxsackievirus A13] AGX00826.1

```
Query  1    CASSQV  6
            CAS+QV
Sbjct  185  CASNQV  190
```

BppU family phage baseplate upper protein [Clostridium perfringens] HBI7084846.1

```
Query  6    VRLAGGGE  13
            VRLAGG E
Sbjct  560  VRLAGGSE  567
```

GIY-YIG nuclease family protein [Clostridium perfringens] WP_164815721.1

```
Query  8   LAGGGEQF  15
           LA GGEQF
Sbjct  85  LAKGGEQF  92
```

myosin XVIIIB, isoform CRA_c [Homo sapiens] EAW59704.1

```
Query  4    SQVRL-AGGGEQ  14
            SQVRL AGGG Q
Sbjct  532  SQVRLPAGGGAQ  543

Query  7     RLAG-GGE  13
             RLAG GGE
Sbjct  2045  RLAGDGGE  2052
```

```
>>sp|P01308|INS_HUMAN Insulin OS=Homo sapiens OX=9606 GN  (
            10
EMBOSS SQVRLAGG
       .::.:.::
sp|P01 GQVELGGG
            70
                  10
EMBOSS CASSQVR---LAGGGEQFF
       :.:  :.   :. : . ::
sp|P01 CGSHLVEALYLVCGERGFF
               40
```

```
>>sp|P47871|GLR_HUMAN Glucagon receptor OS=Homo
              10
EMBOSS ASSQVRLAGG
       .:... ::::
sp|P47 SSAETPLAGG
      460
```

T-cell receptor beta V6-2J1.4, TCRBV6-2J1.4 {complementarity determining region 3} [human, peripheral T-lymphocytes, insulin-dependent diabetes mellitus patient GAD65.05, Peptide Partial, 25 aa] [Homo sapiens] AAB46915.1

```
Query  1   CASS-QVRLAGGGEQFFG  17
           CASS   R A G+E FFG
Sbjct  1   CASSPPGR-ADGDELFFG  17
```

T-cell receptor beta V5-2J2.1, TCRBV5-2J2.1 {complementarity determining region 3} [human, peripheral T-lymphocytes, insulin-dependent diabetes mellitus patient GAD65.05, Peptide Partial, 25 aa] [Homo sapiens] AAB46921.1

```
Query  1   CASSQVRL-AGGG--EQFFG  17
           CASS    L AGGG  EQFFG
Sbjct  1   CASS---LGAGGGGNEQFFG  17
```

TCR diabetic 9: CASSLGQGETEAFFG

polyprotein, partial [Coxsackievirus B3] AAA42932.1
```
Query  6   GQGETE  11
           GQGE+E
Sbjct  8   GQGEIE  13
```

polyprotein, partial [Coxsackievirus B5] ADN93440.1
```
Query  4    SLGQGETEAFF  14
            SL +GET/ +F
Sbjct  214  SLSNGETASMF  224
```

VP1, partial [Coxsackievirus A24] AFV68047.1
```
Query  11  EAFFG  15
           EAFFG
Sbjct  26  EAFFG  30
```

LysM domain-containing protein [Clostridium perfringens] WP_111936546.1
```
Query  3    SSLGQGET  10
            SSLGQG+T
Sbjct  190  SSLGQGDT  197
```

Crystal structure of myosin X motor domain with 2IQ motifs in pre-powerstroke state [Homo sapiens] 5I0I_C
```
Query  4   SLGQGETEA  12
           SLGQ  TEA
Sbjct  37  SLGQNPTEA  45
```

Crystal structure of Myosin VIIa IQ5 in complex with Ca2+-CaM [Homo sapiens] 5WSV_A
```
Query  4   SLGQGETEA  12
           SLGQ  TEA
Sbjct  43  SLGQNPTEA  51
```

T-cell receptor beta V5-2J1.1, TCRBV5-2J1.1 {complementarity determining region 3} [human, peripheral T-lymphocytes, insulin-dependent diabetes mellitus patient GAD65.05, Peptide Partial, 23 aa] [Homo sapiens] AAB46914.1
```
Query  1   CASSLGQGETEAFFG  15
           CASS  \++TEAFFG
Sbjct  1   CASSYEGADTEAFFG  15
```

TCR diabetic 10: CASRNLGLNTEAFFG

polyprotein, partial [Coxsackievirus B5] CCW33423.1
Sequence ID: Length: 1366Number of Matches: 1

```
Query  1    CASRNL  6
            CASRN+
Sbjct  348  CASRNM  353
```

Replication-associated recombination protein A [Clostridium perfringens] MDO4535465.1
Sequence ID: Length: 415Number of Matches: 1

```
Query  6    LGLNTEAFF  14
            L LNTE FF
Sbjct  221  LELNTESFF  229
```

aminotransferase class I/II-fold pyridoxal phosphate-dependent enzyme [Clostridium perfringens] MBO3365597.1

```
Query  2    ASRNLGLNTE  11
            A RNLGLN E
Sbjct  274  ARRNLGLNGE  283
```

```
>>sp|P06213|INSR_HUMAN Insulin receptor OS=Homo sapiens   (
           10
EMBOSS NLGLNTE
       :::: .:
sp|P06 NLGLIEE
           390
           10
EMBOSS NLGLNTEAFFG
       ::... :: .:
sp|P06 NLAAELEANLG
         380

>>sp|Q99259|DCE1_HUMAN Glutamate decarboxylase 1 OS=Homo
               10
EMBOSS CASRNLGLNTEAFFG
       : . :: :...: .:
sp|Q99 CEKYNLWLHVDAAWG
            370
             10
EMBOSS SRNLGLNTEAFF
       .:.:::.  .:.
sp|Q99 TRKLGLKICGFL
      40        50
              10
EMBOSS ASRNLGLNTE
       :.  ::..:.
sp|Q99 AGAALGFGTD
        300
```

TCR diabetic 11: CASSFRRBTDTQYFG

P1, partial [Coxsackievirus A16] AIW00805.1
```
Query  1    CASSFRRBTDT  11
            CAS+ RR+T T
Sbjct  286  CAST-RRDTAT  295
```

AAA family ATPase [Clostridioides difficile] HBY2661863.1
```
Query  6    RRBTDTQY  13
            RR+TD+QY
Sbjct  118  RRDTDNQY  125
```

ethanolamine ammonia-lyase reactivating factor EutA, partial [Clostridioides difficile] WP_269807304.1
```
Query  4   SFRRG--TDTQY  13
           SF+RG  TDTQY
Sbjct  19  SFKRGEVTDTQY  30
```

```
>>sp|Q16849|PTPRN_HUMAN Receptor-type tyrosine-protein p  (
            10
EMBOSS SFRRBTDTQYFG
       .::::...  : :
sp|Q16 DFRRKVNKCYRG
      890       900
```

```
>>sp|Q99259|DCE1_HUMAN Glutamate decarboxylase 1 OS=Homo  (
             10
EMBOSS SSFRRBTDTQY
       . ::: :.:..
sp|Q99 ARFRR-TETDF
          90
```

```
>>sp|P06213|INSR_HUMAN Insulin receptor OS=Homo
               10
EMBOSS CASSFRRBTDTQY
       : .: ::.   ::
sp|P06 CKNS-RRQGCHQY
             300
```
myosin XVI [Homo sapiens] KAI2569880.1
```
Query  9    TDTQYF  14
            TD+QYF
Sbjct  714  TDIQYF  719
```

TCR DIA K2.12 CASSSDRLGNQPQHF

polyprotein, partial [Coxsackievirus A10] CCC55386.1

```
Query  2   ASSSDRLG  9
           A SS RLG
Sbjct  33  APSSHRLG  40
```

hypothetical protein BN182_1230007 [Clostridioides difficile E9] CCL59956.1

```
Query  8   LGNQPQHF  15
           LGNQP HF
Sbjct  31  LGNQPLHF  38
```

Restriction endonuclease [Clostridium perfringens] EHK2335059.1

```
Query  2    ASSSDRLGNQP  12
            ASSSDR G QP
Sbjct  205  ASSSDRRG-QP  214
```

VPA1262 family protein [Clostridium perfringens] MDM0720080.1

```
Query  4    SSDRLGN  10
            SS+RLGN
Sbjct  160  SSERLGN  166
```

unconventional myosin-Id isoform 4 [Homo sapiens] NP_001398017.1

```
Query  7    RLGNQPQ  13
            RL NQPQ
Sbjct  896  RL-NQPQ  901
```

myosin ID, isoform CRA_c [Homo sapiens] EAW80223.1

```
Query  7    RLGNQPQ  13
            RL NQPQ
Sbjct  973  RL-NQPQ  978
```

calcium-transporting ATPase type 2C member 2 isoform X2 [Homo sapiens] XP_047290950.1

```
Query  4    SSDRLGNQ  11
            S DRLG+Q
Sbjct  267  SMDRLGKQ  274
```

```
>>sp|P06213|INSR_HUMAN Insulin receptor OS=Homo sapiens
           10
EMBOSS SDRLGNQPQ
       :. :::  :
sp|P06 SECLGNCSQ
        240
```

```
>>sp|P01275|GLUC_HUMAN Pro-glucagon OS=Homo sapiens OX=9
              10
EMBOSS ASSSDRLGNQPQ
       ::..: :.. /:
sp|P01 ASQADPLSDPDQ
           40
```

```
>>sp|Q99259|DCE1_HUMAN Glutamate decarboxylase 1 OS=Homo  (
             10
EMBOSS  SSSDRLGNQPQ
        :..   .: :::
sp|Q99  SGTTMVGYQPQ
       550
```

T-cell receptor beta V23-2J1.5, TCRBV23-2J1.5 {complementarity determining region 3} [human, peripheral T-lymphocytes, insulin-dependent diabetes mellitus patient GAD65.05, Peptide Partial, 25 aa] [Homo sapiens] AAB46923.1

```
Query  1    CASS-SDR-LGNQPQHF  15
            CASS   DR +G QPQHF
Sbjct  1    CASSLWDRGMG-QPQHF  16
```

TCR DIA K2.16 CASSQDRLRGVADTQYF

VP1, partial [Coxsackievirus A6] QGF19190.1

```
Query  6     DRLRGVADTQY  16
             DRLR   A+T+Y
Sbjct  284   DRLRS-ATTDY  293
```

hypothetical protein, partial [Clostridium perfringens] WP_198621907.1

```
Query  2    ASSQDRLRGVA  12
            ASS DRLR   A
Sbjct  5    ASSMDRLRAAA  15
```

N-acetylglucosamine-6-phosphate deacetylase [Clostridium perfringens] NWJ14636.1

```
Query  3     SSQDRLRGVADT  14
             SS +RL   VA+T
Sbjct  98    SSKERLKDVAET  109
```

type I restriction endonuclease subunit R [Clostridium perfringens] HAT4325355.1

```
Query  6     DRLRGVAD  13
             DRLR+VA+
Sbjct  525   DRLRAVAE  532
```

DUF975 family protein [Clostridium perfringens] WP_265869693.1

```
Query  4    SQDRLRG  10
            SQD+LRG
Sbjct  10   SQDKLRG  16
```

Diacylglycerol O-acyltransferase [Clostridioides difficile] VTR08062.1

```
Query  2     ASSQ---DRLRGVAD  13
             AS Q    DRLR VAD
Sbjct  242   ASQQWEIDRLRAVAD  256
```

ftsX-like permease family protein [Clostridioides difficile P28] EQJ57339.1

```
Query  8    LRGVADTQY  16
            LRGV DT+Y
Sbjct  443  LRGVDDTEY  451
```

ABC transporter permease [Clostridioides difficile] MDB3323854.1

```
Query  8    LRGVADTQY  16
            LRGV DT+Y
Sbjct  443  LRGVDDTEY  451
```

```
>>sp|Q99259|DCE1_HUMAN Glutamate decarboxylase 1 OS=Homo  (
        10
EMBOSS LRGVADT
       :::: :.
sp|Q99 LRGVPDS

>>sp|P06213|INSR_HUMAN Insulin receptor OS=Homo sapiens
         10
EMBOSS RLRGVADTQY
       ::::..  .:
sp|P06 RLRGLSPGNY
             920
              10
EMBOSS ASSQDRLRGVADTQ
       ....: ..: :.:.
sp|P06 GNARDIIKGEAETR
    1040      1050
```

TCR DIA K2.4 CASSQEADIQYF

polyprotein [Coxsackievirus B5] UWT50257.1

```
Query  3    SSQE-ADIQ  10
            SSQE  +IQ
Sbjct  692  SSQEQSTIQ  700
```

polyprotein [Coxsackievirus B4] APR72754.1

```
Query  3     SSQE---ADIQYF  12
             S+QE    +IQYF
Sbjct  1168  SDQEQLFSNIQYF  1180
```

polyprotein [Coxsackievirus B5] AVJ35737.1

```
Query  3     SSQE---ADIQYF  12
             S QE    +IQYF
Sbjct  1171  SDQEQLFSNIQYF  1183
```

polyprotein, partial [Coxsackievirus B3] UPI41529.1
```
Query  3     SSQE---ADIQYF  12
             S QE    +IQYF
Sbjct  1171  SDQEQLFSNIQYF  1183
```

polyprotein [Coxsackievirus B2] ANQ31694.1
```
Query  3     SSQE---ADIQYF  12
             S QE    +IQYF
Sbjct  1173  SDQEQLFSNIQYF  1185
```

excinuclease ABC subunit UvrA [Clostridioides difficile] NJJ35117.1
```
Query  7    ADIQYF  12
            ADIQYF
Sbjct  287  ADIQYF  292
```

beta-ketoacyl-ACP synthase II [Clostridioides difficile] HBF4251760.1
```
Query  5    QEADIQY  11
            QEADI+Y
Sbjct  382  QEADIEY  388
```

hypothetical protein [Clostridium perfringens] MDK0914316.1
```
Query  2    ASSQEADIQY  11
            A+SQE +IQY
Sbjct  194  ATSQEKQIQY  203
```

recombinase family protein [Clostridium perfringens] WP_075809589.1
```
Query  3    SS-QEADIQYF  12
            SS QE DIQ+F
Sbjct  445  SSYQE-DIQFF  454
```

```
>>sp|Q16849|PTPRN_HUMAN Receptor-type tyrosine-protein p
                10
EMBOSS CASSQ-EADIQ
       ::..: :..:.
sp|Q16 CATAQGEGNIK
         730
```

TCR Heart 2.1 CASSQDVAGLGFFG

Polyprotein, Coxsackievirus A13, WAB71078.1

```
TCR Heart 2.1            3    SSQDVAGLGFFG  14
                              SSQD AG +++G
CoxA13                 805    SSQD-AGAAYYG  815
```

Spore germination protein YndE, Streptococcus pneumoniae, CKI08002.1
hypothetical protein, Streptococcus anginosus, MCW1043024.1

```
TCR Heart 2.1             7   VAGLGFFG  14
                              VAG+GFFG
S.pneumoniae             57   VAGIGFFG  64
S.anginosus              66   IAGLGFF  72
```

ABC transporter ATP-binding protein, Streptococcus sanguinis, WP_002923689.1

```
TCR Heart 2.1             4    SQDVAGL  10
                               SQDVAGL
S.sanguinis             220    SQDVAGL  226
```

tail fiber domain-containing protein, Streptococcus pneumoniae, MDG7249185.1

```
TCR Heart 2.1             4   SQDVAGLGFFG  14
                              SQDVA   FFG
S.pneumoniae             35   SQDVA--NFFG  43
```

myosin XVA, isoform CRA_b, Homo sapiens, EAW55664.1
unconventional myosin-XV isoform X4, Homo sapiens, XP_024306549.1

```
Heart 2.1                 5    QDVAGLG  11
                               Q VAGLG
Myosin                  551    QAVAGLG  557
Unconv. Myosin         1992    QAVAGLG  1998
```

laminin, alpha 1, isoform CRA_d, partial, Homo sapiens, EAX01631.1

```
Laminin                2028   DVAGL  2032
                              DVAGL
Heart 2.1                 6   DVAGLGFF  13
                              DV GL F+
Laminin                2831   DVEGL-FY  2837
```

collagen type IV alpha 2 chain, Homo sapiens, KAI2569886.1

```
Collagen               1116   GLKGFFG  1122
                              GL GFFG
TCR Heart 2.1             9   GL-GFFG  14
TCR Heart 2.1             9    GLGFFG  14
                               GLGF+G
Collagen                232    GLGFYG  237
```

Chain C, Troponin I, Homo sapiens, 1J1E_C
Chain U, Troponin I, cardiac muscle, Homo sapiens, 6KN7_U

```
TCR Heart 2.1             8   AGLGF  12
                              AGLGF
Troponin                 46   AGLGF  50
```

insulin isoform U2 [Homo sapiens] QMS45322.1
Sequence ID: Length: 198Number of Matches: 1

```
Query  3   SSQDVAGL  10
           S +DVAGL
Sbjct  54  SREDVAGL  61
```

TCR Heart 2.2 CASSQDVAGLAFFG (2 clones)

```
TCR Heart 2.2                  4    SQDVAGL  10
                                    SQDVAGL
S.sanguinis                  220   SQDVAGL  226
```
ABC transporter ATP-binding protein **Streptococcus sanguinis**, WP_004192995.1

DUF1275 family protein **Streptococcus agalactiae**, MCD0152263.1

```
S.agal.                       68   VAGLAFF  74
                                   VAGLAFF
TCR Heart 2.2                  7   VAGLAFFG  14
                                   VA LAFFG
S.sanguinis                            54   VASLAFFG  61
```
type II CAAX endopeptidase protein **Streptococcus sanguinis**, WP_223313500.1

```
TCR Heart 2.20                 5   QDVAGLAFF  13
                                   Q +AGLAFF
S.pneumoniae                  42   QGAAGLAFF  50
```
Uncharacterised Protein (DUF2627) **Streptococcus pneumoniae**,COC68514.1

```
TCR Heart 2.2                  7   VAGLA-FFG  14
                                   VAGLA FFG
S.pneumoniae                  12   VAGLAVFFG  20
```
sugar ABC transporter permease **Streptococcus pneumoniae**, COI72814.1

```
TCR Heart 2.2                  3    SSQDVAGLAFFG  14
                                    SSQD AG A++G
CoxA13                       805   SSQD-AGAAYYG  815
```
polyprotein **Coxsackievirus A13**, WAB71078.1

collagen alpha-1(XVII) chain, Homo sapiens, NP_000485.3

```
Heart 2.2                      2    ASSQDVAG  9
                                    ASSQ+V+G
Collagen                     198   ASSQSVSG  205
```

plasma membrane calcium-transporting ATPase 2 isoform 3, Homo sapiens, NP_001317540.1

```
Heart 2.2                      4    SQDVAGL  10
                                    SQDVA L
Ca+ transp.                 1122   SQDVANL  1128
```

ryanodine receptor 1 isoform X4, Homo sapiens, XP_047295158.1

```
Heart 2.2                      5     QDVAGLAFF  13
                                     QDV+ L FF
```

```
Ryanodine rec.                1205  QDVSSLRFF  1213
```

T cell receptor beta chain, partial [Homo sapiens] AAG15724.1

```
Query  1   CASSQDVAG  9
           CASSQDV G
Sbjct  10  CASSQDVQG  18

Slachta,C.A., Jeevanandam,V., Goldman,B., Lin,W.L. and
            Platsoucas,C.D.
  TITLE     Coronary arteries from human cardiac allografts with chronic
            rejection contain oligoclonal T cells: persistence of identical
            clonally expanded TCR transcripts from the early
            post-transplantation period (endomyocardial biopsies) to chronic
            rejection (coronary arteries)
  JOURNAL   J. Immunol. 165 (6), 3469-3483 (2000)
```

T cell receptor beta chain, partial [Homo sapiens] BAC01027.1

```
Query  1   CASSQDYRAADTRFFG  16
           CASSQD  +ADT +FG
Sbjct  3   CASSQD-SGADTQYFG  17

Saito,M., Higuchi,I., Saito,A., Izumo,S., Usuku,K., Bangham,C.R.
            and Osame,M.
  TITLE     Molecular analysis of T cell clonotypes in muscle-infiltrating
            lymphocytes from patients with human T lymphotropic virus type 1
            polymyositis
  JOURNAL   J. Infect. Dis. 186 (9), 1231-1241 (2002)
```

C4-dicarboxylate-binding periplasmic protein, Streptococcus pneumoniae], CJL99217.1

```
TCR Heart 2.3                 2    ASSQRFQ----GRA  11
                                   A++QRFQ    GRA
S.pneumoniae                  116   AAAQRFQQSDKGRA  129
```

T-cell receptor beta V17-1J1.2, TCRBV17.1J1.2 {complementarity determining region 3} [human, peripheral T-lymphocytes, insulin-dependent diabetes mellitus patient GAD65.05, Peptide Partial, 21 aa] [Homo sapiens]
Sequence ID: AAB46922.1Length: 21Number of Matches: 1
Range 1: 1 to 13GenPeptGraphics Next Match Previous Match

Alignment statistics for match #1

Score	Expect	Identities	Positives	Gaps
20.2 bits(40)	481	10/15(67%)	10/15(66%)	3/15(20%)

```
Query  1   CASSQRFQGRAY-FG  14
           CASS+R QG +Y FG
Sbjct  1   CASSNR-QG-GYTFG  13
```

TCR Heart 2.4 CASSALRGVYFFG (1 clone)

polyprotein, partial, Coxsackievirus B5, CCW33418.1

```
CoxB5 & CoxB4                    1143  SAALLR-HYF  1151
                                        S+ALR  YF
TCR Heart 2.4                    1     CASSALRGVYF  11
                                       CA+    GVYF
CoxB5 & CoxB4                    341   CAT----GVYF  347
```

polyprotein, partial, Coxsackievirus B4, AAV69735.1

polyprotein, partial, Coxsackievirus A9, AAF27790.1
polyprotein, partial, Coxsackievirus A13, CAC08130.1

```
TCR Heart 2.4                   1    CASSALRGVYF  11
                                     CA+ A  GVYF
CoxA9 & CoxA13                  173  CACRA--GVYF  181
```

homoserine O-succinyltransferase, Streptococcus pneumoniae, MXQ48449.1
homoserine O-succinyltransferase, Streptococcus agalactiae, WP_017769226.1

```
TCR Heart 2.4                    2    ASSALRGVYFFG  13
                                      ASS LR VY FG
S.pneumoniae & S. agal.                  223  ASSDLREVYSFG  234
```

PolC-type DNA polymerase III [Streptococcus pyogenes] WP_136307060.1

```
TCR Heart 2.4                    5     ALRGVYF  11
                                        ALRG+YF
S.pyogenes                               1298  ALRGAYF  1304
```

alpha/beta hydrolase-fold protein, Streptococcus pneumoniae, WP_221073517.1

```
TCR Heart 2.4                     5    ALRGVY---FF  12
                                        ALRGVY   FF
S.pneumoniae                      168  ALRGVYDARFF  178
```

myosin IG, isoform CRA_e, Homo sapiens, EAW61072.1
unconventional myosin 1G valine form, partial, Homo sapiens, AAK58092.1

```
TCR Heart 2.4                    5    EGF---DRDHLF  13
                                      EGF   +RD+LF
Unoonv. Myosin                   497  EGFIDKNRDFLF  508
```

actin filament-associated protein 1 isoform X5, Homo sapiens, XP_016864025.1

```
TCR Heart 2.4                    3   SSEGFD-RDH  11
                                      SS GFD +DH
Actin-assoc.                     40  SSKGFDVKDH  49
```

glutamate receptor, metabotropic 8, isoform CRA_d, Homo sapien, EAW83624.1

```
TCR heart 2.4                    2    ASSEGFDR  9
                                       AS +GFDR
Glut Rec                         336  ASIDGFDR  343
```

laminin subunit alpha-5 isoform X6, Homo sapiens, XP_054179422.1

```
TCR Heart 2.4                    1   CASSAL--RG  8
                                     CA SAL  RG
Laminin                          6   CAGSALCVRG  15
```

myosin-reactive immunoglobulin heavy chain variable region, partial [Homo sapiens]
AAD56259.1

```
Query  1    CA---SSEGFD  8
            CA   SSE+FD
Sbjct  96   CARGDSSEAFD  106
```

TCR Heart 2.5 CASSEGFDRDHLFG (1 Clone)

Asp-tRNA(Asn)/Glu-tRNA(Gln) amidotransferase subunit GatC, Streptococcus pneumoniae, WP_088834616.1

```
TCR Heart 2.5                  5    EGFDRDHLF  13
                                     EG DRDHLF
S.pneumoniae                   69   EGIDRDHLF  77
```

NAD-dependent deacetylase, Streptococcus pneumoniae, VTQ32396.1

```
TCR Heart 2.5                  2    ASSEGFDRD  10
                                     A+ EGFDRD
S.pneumoniae                   76   ATPEGFDRD  84
```

Predicted integral membrane protein, Streptococcus pneumoniae, COE84929.1

```
TCR Heart 2.5                  5    EGFDRDHLF  13
                                    EG++RD+LF
S.pneumoniae                   37   EGYERDQLF  45
```

hypothetical protein , Streptococcus agalactiae, WP_001255838.1
hypothetical protein, Streptococcus pyogenes, WP_136107756.1

```
TCR Heart 2.5                  4    SEGFDRDHL  12
                                    SEG D+DHL
S.pyogenes & S.agal.           71   SEGLDKDHL  79
```

LytTR family DNA-binding domain-containing protein, Streptococcus sanguinis, WP_176798752.1

```
TCR Heart 2.5                  6    GFDRDH--LF  13
                                    GFDR+H  LF
```

```
S.sanguinis                       75  GFDREHKTLF  84
```

myosin IG, isoform CRA_e, Homo sapiens, EAW61072.1

```
TCR Heart 2.5                     5    EGF---DRDHLF  13
                                       EGF   +RD+LF
Myosin                            291  EGFIDKNRDFLF  302
```

myosin ID, isoform CRA_e, partial, Homo sapiens, EAW80225.1

```
TCR Heart 2.5                     1    CASS---EGFDRD  10
                                       CAS+   E FDRD
Myosin                            506  CASDKILE-FDRD  517
```

actin filament-associated protein 1 isoform X5, Homo sapiens, XP_016864025.1

```
TCR Heart 2.5                     3   SSEGFD-RDH  11
                                      SS GFD +DH
Actin filament                    40  SSKGFDVKDH  49
```

Chain A, Metabotropic glutamate receptor 8, Homo sapiens, 6BSZ_A

```
TCR Heart 2.5                     2    ASSEGFDR  9
                                       AS +GFDR
Glutamate rec.                    300  ASIDGFDR  307
```

acetylcholine-specific T-cell receptor beta chain, partial [Homo sapiens] AAB25007.1

```
Query  1   CASS-EGF  7
           CASS EG+
Sbjct  4   CASSLEGY  11
```

myosin-reactive immunoglobulin heavy chain variable region, partial [Homo sapiens] AAD56259.1

```
Query  1    CA---SSEGFD  8
            CA   SSE+FD
Sbjct  96   CARGDSSEAFD  106
```

TCR Heart 2.6 CASSQGLGYTFFG (1 CLONE)

VP1, partial, Coxsackievirus A16, AIW00798.1

```
CoxA16                          34   ASSHGLG  40
                                     ASS+GLG
TCR Heart                  2.6  2    ASSQGLG  8
                                     ASSQGL
CoxB1                           329  ASSQGL   334
```

Polyprotein, Coxsackievirus B1, ABV64405.1

polyprotein, partial, Coxsackievirus A11, WAB71058.1
polyprotein, partial, Coxsackievirus A15, BAZ95882.1
polyprotein, partial, Human enterovirus, QRG33074.1

```
TCR Heart 2.6                   2    ASSQGLGYT  10
                                      AS+Q LGYT
CoxA11 & CoxA15                 430  ASEQRLGYT  438
```

putative arabinose transporter, Streptococcus pneumoniae, CJR70294.1
Purine efflux pump PbuE, Streptococcus pneumoniae, CVN64787.1
Sequence ID: Length: 396Number of Matches: 1

```
TCR Heart 2.6                    5    QGLGYTFFG  13
                                      +GLGY+FFG
S.pneumoniae                     245  HGLGYVFFG  253
```

LLM class flavin-dependent oxidoreductase, Streptococcus pyogenes, WP_136274362.1

```
TCR Heart 2.6                   6      GLGYTF  11
                                       GLGYTF
S.pyogenes                               177  GLGYTF  182
```

myosin XV variant, partial, Homo sapiens, BAD92660.1

```
TCR Heart 2.6                   3    SSQGLGY  9
                                     SS GLGY
Myosin                          659  SSPGLGY  665
```

glutamate ionotropic receptor NMDA type subunit 2A, partial, Homo sapiens, KAI2577129.1

```
TCR Heart 2.6                   2    ASSQGL-GYTFF  12
                                     A S GL GY+FF
Glut. Rec.                      107  ARSLGLTGYDFF  118
```

axonemal dynein, partial, Homo sapiens, AAC27542.1

```
TCR Heart 2.6                   4   SQG--LGYTF  11
                                    SQG  LGYTF
Dynein                          86  SQGRKLGYTF  95
```

TCR Heart 2.7 CASSQDRGQGFFFG (1 CLONE)

radical SAM peptide maturase, CXXX-repeat target family, Streptococcus pneumoniae, MDG8229751.1

```
TCR Heart 2.7                 1    CASSQDR  7
                                    CASS+DR
S.pneumoniae                  411   CASSEDR  41
```

zinc metalloprotease ZmpD, Streptococcus pneumoniae, WP_000054783.1

```
TCR Heart 2.7                 6    DRGQGF  11
                                    DRGQGF
S.pneumoniae                  452  DRGQGF  457
```

bacteriocin immunity protein, Streptococcus pneumoniae, WP_061756112.1

```
TCR Heart 2.7                 2   ASSQDRG  8
                                  ASSQ+RG
S.pneumoniae                  78  ASSQERG  84
```

hypothetical protein, Streptococcus pyogenes, WP_136022607.1

```
TCR Heart 2.7                 2    ASSQ-DRG-QG  10
                                    ASSQ DRG QG
S.pyogenes                    159  ASSQGDRGHQG  169
```

GHKL domain-containing protein, Streptococcus agalactiae, WP_047201031.1

```
TCR Heart 2.7                 2   ASS--QDRGQGFF  12
                                   ASS  +DRG GF+
S.agalactiae                  56  ASSKGEDRGLGFY  68
```

filamin A, alpha (actin binding protein 280), isoform CRA_d,,Homo sapiens, EAW72747.1

```
TCR Heart 2.7                 5     QDRGQG  10
                                     QDRG+G
Filamin                       1310  QDRGDG  1315
```

unconventional myosin-VIIb isoform X5, Homo sapiens, XP_016859658.1

```
TCR Heart 2.7                 4     SQ----DRGQG  10
                                     SQ    DRGQG
Mysoin                        1435  SQVMASDRGQG  1445
```

T-cell receptor beta V12-1J2.6, TCRBV12-1J2.6 {complementarity determining region 3} [human, peripheral T-lymphocytes, insulin-dependent diabetes mellitus patient GAD65.05, Peptide Partial, 28 aa] [Homo sapiens] AAB46918.1

```
Query  1  CASSQDRGQG  10
          CASS + GQG
Sbjct  1  CASSVEPGQG  10
```

T cell receptor beta chain, partial [Homo sapiens] BAC01026.1

```
Query  1   CASSQDRGQ--GFFFG  14
           CASSQDRGQ  G+ FG
Sbjct  3   CASSQDRGQVYGYTFG  18
```

Saito,M., Higuchi,I., Saito,A., Izumo,S., Usuku,K., Bangham,C.R.
            and Osame,M.

```
  TITLE     Molecular analysis of T cell clonotypes in muscle-infiltrating
            lymphocytes from patients with human T lymphotropic virus type 1
            polymyositis
  JOURNAL   J. Infect. Dis. 186 (9), 1231-1241 (2002)
```

T cell receptor beta chain, partial [Homo sapiens] AAG15721.1

```
Query  1   CASSQDRGQGFFFG  14
           CASSQ RG+   FFG
Sbjct  10  CASSQGRGE-LFFG  22

Slachta,C.A., Jeevanandam,V., Goldman,B., Lin,W.L. and
            Platsoucas,C.D.
Coronary arteries from human cardiac allografts with chronic
            rejection contain oligoclonal T cells: persistence of identical
            clonally expanded TCR transcripts from the early
            post-transplantation period (endomyocardial biopsies) to chronic
            rejection (coronary arteries)
  JOURNAL   J. Immunol. 165 (6), 3469-3483 (2000)
```

TCR Heart 2.8 CASSQDGVANQRSFFG (1 CLONE)

curlin minor subunit CsgB, Streptococcus pneumoniae, CJK66416.1

```
TCR Heart 2.8                   4    SQDGVANQ  11
                                     SQDGV NQ
S.pneumoniae                    142  SQDGVSNQ  149
```

alpha/beta hydrolase-fold protein, partial, Streptococcus sanguinis, WP_142999620.1

```
TCR Heart 2.8                   8    VANQRSFFG  16
                                     V+NQRS FG
S.sanguin.                      56   VTNQRSIFG  64
```

short-chain dehydrogenase oxidoreductase, Streptococcus pneumoniae, CGF85861.1

```
TCR Heart 2.8                   7    GVANQRS  13
                                     GVANQRS
S.pneumoniae                    13   GVANQRS  19
```

Eco57I restriction-modification methylase domain-containing protein, Streptococcus anginosus, WP_264339775.1

```
TCR Heart 2.8                   3    SSQDGVANQ  11
                                     SS+DGVAN+
S.anginosus                     606  SSDDGVANE  614
```

Tfp pilus assembly protein FimV,Streptococcus pneumoniae, CJL37375.1

```
TCR Heart 2.8                   2    ASSQDGVANQ  11
                                     ASS+ GVAN+
S.pneumoniae                    402  ASSEGGVANE  411
```

Uncharacterised protein, Streptococcus pneumoniae, COQ53279.1

```
TCR Heart 2.8                    4    SQDGVANQRSF  14
                                      SQDGV +QR F
S.pneumoniae                     505  SQDGVFEQRHF  515
```

Dynein axonemal heavy chain 5 isoform X7, Homo sapiens, XP_054207919.1

```
Dynein                          3189  QDGVA  3193
                                      QDGVA
TCR Heart 2.8                    5    QDGVA  9
                                      QDGVA
Myosin                           656  QDGVA  660
```

human Myosin 1c, Homo sapiens, 4BYF_A

Laminin, alpha 1, isoform CRA_a, partial, Homo sapiens, EAX01628.1

```
TCR Heart 2.8                    3     SSQDGV  8
                                        SSQ+GV
Laminin                          2674  SSQNGV  2679
```

Laminin subunit alpha-5 isoform X7, Homo sapiens, XP_054179424.1

```
TCR Heart 2.8                    1    CASSQDGVANQRSFFG  16
                                      CAS++DG      FFG
Laminin                          808  CASCKDG------FFG  817
```

TCR Heart 2.9 CASSATGAGTGEFFG (1 CLONE)

polyprotein, partial, Coxsackievirus B4, WDE31181.1

```
TCR Heart 2.9                    2   AS-SATG  7
                                     AS SATG
CoxB4                            15  ASLSATG  21
```

dihydrodipicolinate synthase, Streptococcus pneumoniae, CJL82484.1
dihydrodipicolinate synthase family protein, Streptococcus agalactiae, MCQ3823083.1

```
TCR Heart 2.9                    2   ASS--ATGAGTGEFF  14
                                     AS   A+G GTGEFF
S.pneumoniae                     5   ASALFAAG-GTGEFF  18
```

Serine transporter, Streptococcus pneumoniae, COE01047.1

```
TCR Heart 2.9                    2    ASSAT-G--AGTGEFFG  15
                                      A SAT G  A TGE+FG
S.pneumoniae                     87   AGSATDGILAVTGEYFG  103
```

portal protein, Streptococcus pneumoniae, VSZ18930.1

```
TCR Heart 2.9                    6    TGAGTGE  12
                                      TGAGTGE
S.pneumoniae                     174  TGAGTGE  180
```

anaerobic ribonucleoside triphosphate reductase, Streptococcus pneumoniae, CJL47967.1

```
TCR Heart 2.9                    5   ATGAGTGEF  13
                                     A+GA+TGEF
S.pneumoniae                     34  AAGAATGEF  42
```

myosin ID, isoform CRA_b, partial, Homo sapiens, EAW80222.1

```
TCR Heart 2.9                    3    SSATGAGTGEFFG  15
                                      SSATGA     FG
Myoson                           950  SSATGA-----FG  957

TCR Heart 2.9                    7    GAGTGE  12
                                      GAGT+E
Myosin                           440  GAGTAE  445
```

filamin A, alpha (actin binding protein 280), isoform CRA_d, Homo sapiens, EAW72747.1

```
TCR Heart 2.9                    7    GAGTGE  12
                                      GAGTGE
Filamin                          410  GAGTGE  415

TCR Heart 2.9                    7    GAGTGE  12
                                      GAG+GE
Filamin                          509  GAGSGE  514
```

TCR Heart 2-10 CASSQDYRAADTRFFG (1 clone)

VP1 protein, partial, Coxsackievirus A13, CAD98887.1, M1Q4H9;
Coxsackie virus A18 B2X7L9

```
TCR Heart 2-10:                   6   DYRAAD   11
                                      DY+AAD
CoxA13, CoxA18                   84   DYKAAD   89
```

polyprotein, partial, Coxsackievirus A13, UBM82731.1

```
TCR Heart 2.10                   2    ASSQDYR  8
                                      ASS+DYR
CoxA13                           286  ASSEDYR  292
```

```
Polyprotein, Coxsackievirus A6, AVQ94429.1
TCR Heart 2.10                  2    ASSQDYRAAD-TRF  14
                                     AS QD+ A D T+F
CoxA6                           41   ASRQDF-AQDPTKF  53
```

chorismate binding enzyme para-aminobenzoate synthetase, Streptococcus pneumoniae, CJD24034.1

```
TCR Heart 2.10                  1     CASSQDYR  8
                                      CA +QDYR
S.pneumoniae                    425   CAANQDYR  432
```

tRNA 2-selenouridine synthase, Streptococcus pneumoniae, VTQ31256.1

```
TCR Heart 2.10                  5    QDYRA---ADT  12
                                     QDYRA   ADT
S.pneumoniae                    8    QDYRALLIADT  18
```

single-stranded DNA-binding protein, Streptococcus anginosus, RGY85239.1

```
TCR Heart 2.10                  2    ASSQDYRAAD  11
                                     A+S DY+AAD
S.anginosus                     31   ATSRDYKAAD  40
```

hypothetical protein, partial, Streptococcus pyogenes, WP_216353496.1

```
TCR Heart 2.10                  2    ASSQD----YRAADTR  13
                                     A S+D    YRAA+TR
S.pyogenes                          61   AASDDFASRYRAAQTR  76
```

unc-45 myosin chaperone B, Homo sapiens, KAI4048879.1

```
TCR Heart 2.10                  5    QDYRAA  10
                                     QDY+AA
Myosin chaperone                20   QDYKAA  25
```

collagen type VI alpha 3 chain, partial, Homo sapiens, KAI2527606.1

```
TCR Heart 2.10                  3     SSQDYRAAD  11
                                      SS  YRAA+
Collagen VI                     539   SSAGYRAAE  547
```

outer dynein arm-docking complex subunit 2 isoform 2, Homo sapiens, NP_001276950.1

```
TCR Heart 2.10                  1     CASSQDYRAA  10
                                      CAS+++YRAA
Dynein                          184   CASEENYRAA  193
```

TCR Heart 2-11 CASSQDRGGGAGFG (1 clone)

polyprotein, partial, Coxsackievirus A1, UBM82506.1
```
CoxA1                         424   GAGFG  428
                                    GAGFG
TCR Heart 2.11                  8   GGGAGFG  14
                                    GGGAG
CoxA20                          1   GGGAG  5
```
polyprotein, partial [Coxsackievirus A20] AEG42513.1

molecular chaperone DnaJ, Streptococcus pneumoniae, MTV44814.1
phage portal protein, lambda family [Streptococcus pneumoniae] VTQ34062.1, 23-29
```
S.pneumoniae                   5        GGGAGFG  11
                                         GGGAGFG
TCR Heart 2.11                  4    SQDRGGGAGFG  14
                                     SQD+   GAGFG
S.pneumoniae                  303   SQDKNQGAGFG  313
```
ribonucleotide-diphosphate reductase subunit alpha, Streptococcus pneumoniae, CKH96652.1

radical SAM peptide maturase, CXXX-repeat target family, Streptococcus pneumoniae, MDG8229751.1
putative transcriptional regulator, Streptococcus pneumoniae, VSE37131.1
```
TCR Heart 2.11                  1    CASSQDR   7
                                     CASS+DR
S.pneumoniae                  411   CASSEDR   417
```

Transcription termination factor Rho, Streptococcus pneumoniae, CVM18608.1
```
S.pneumoniae                  134   ASDQDRSGG  143
                                    AS+QDR GG
TCR Heart 2.11                  2   ASSQDRGGG  9
                                    ASSQ+RG
S.pneumoniae                 1598    ASSQNRG    1607
```
phage hyaluronidase. Streptococcus pneumoniae, CVQ11983.1

amino acid ABC transporter periplasmic protein, Streptococcus pneumoniae, CJK97637.1
```
TCR Heart 2.11                  5   QDRGGGAGF  13
                                    QDR G AGF
S.pneumoniae                   56   QDRNGEAGF  64
```

unconventional myosin-VIIb isoform X1-8, Homo sapiens. XP_047300396.1
```
TCR Heart 2.11                  4    SQ----DRGGGAGFG  14
                                     SQ    DRG GAG+G
S.pneumoniae                  259   SQVMASDRGQGAGMG  273
```

F-actin-uncapping protein LRRC16A isoform X17, Homo sapiens, XP_054211808.1
```
TCR Heart 2.11                  3     SSQDR--GGGAG 14
                                      SS +R   GGGAG
S.pneumoniae                 1181    SSVERSDGGGAG  1199
```

myosin light chain kinase 2, skeletal muscle, isoform CRA_d, Homo sapiens, EAW76417.1
```
TCR Heart 2.11                  3   SSQ------DRGGG  10
                                    SSQ      DRGGG
S.pneumoniae                   72   SSQGPKGEGDRGGG  85
```

myosin-reactive immunoglobulin heavy chain variable region, partial [Homo sapiens] AAD56255.1

```
Query  1     CASSQDRGGG  10
             CA SQ  GGG
Sbjct  96    CARSQ--GGG  103
```

T cell receptor beta chain, partial [Homo sapiens] BAC01026.1

```
Query  1  CASSQDRG  8
          CASSQDRG
Sbjct  3  CASSQDRG  10
```

Saito,M., Higuchi,I., Saito,A., Izumo,S., Usuku,K., Bangham,C.R. and Osame,M.
TITLE Molecular analysis of T cell clonotypes in muscle-infiltrating lymphocytes from patients with human T lymphotropic virus type 1 polymyositis
JOURNAL J. Infect. Dis. 186 (9), 1231-1241 (2002)

TCR Heart 2-12 CASSQDYRAAYFG (2 clones)

polyprotein, Coxsackievirus A13, URT60091.1

```
TCR Heart 2.12                  2   ASSQDY  7
                                    A+SQDY
TCR Heart 2.12                  2   ASSQDYR  8
                                    ASS+DYR
CoxA13                        286   ASSEDYR  292
```

polyprotein, partial, Coxsackievirus A13, Sequence ID: UBM82731.1

Polyprotein, Coxsackievirus A13] WAB71078.1

```
TCR Heart 2.12                3   SSQDYRAAYFG  13
                                  SSQD  AAY+G
CoxA13                      805   SSQDAGAAYYG  815

TCR Heart 2-12                 1    CASSQDYR  8
                                    CA++QDYR
S.pneumoniae                 425    CAANQDYR  432
```

aminodeoxychorismate synthase component I, Streptococcus pneumoniae , WP_050282022.1

```
TCR Heart 2-12                4    SQDYRA-----AYFG  13
                                   SQD+RA     AYFG
S.anginosus                 197    SQDHRAAIAFLAYFG  211
```

ABC transporter ATP-binding protein **Streptococcus anginosus** WP_223351257.1

```
TCR Heart 2-12                  5   QDY-RAAYF  12
                                    +DY RAAYF
S.pyogenes & S.agal.           15   EDYERAAYF  23
```
AAA family ATPase, partial Streptococcus pyogenes, WP_031499689.1
ATP-dependent Clp protease, ATP-binding protein ClpE, Streptococcus agalactiae, VFA67960.1, 32-40

outer dynein arm-docking complex subunit 2 isoform X1, Homo sapiens, XP_024303817.1
Sequence ID: Length: 1107Number of Matches: 1
```
TCR Heart 2.12                    1    CASSQDYRAA  10
                                       CAS+++YRAA
Dynein                          659    CASEENYRAA  668
```

unc-45 myosin chaperone B, Homo sapiens, KAI4048880.1
```
TCR Heart 2.12                    5   QDYRAA  10
                                      QDY+AA
Myosin chaperone                 20   QDYKAA  25
```

TCR Heart 2.13 CASSPRTGRYEQYF (6 clones)

VP1, partial Coxsackievirus A16, QNL13216.1
```
TCR Heart 2.13                  2   ASSPRTG  6
                                    ASSPR G
CoxA16                         34   ASSPRLG  42
```

VP1, Coxsackievirus A14, AAD40501.1
```
CoxA14                        121   CASSSRT  127
                                    CASS RT
TCR Heart 2.13                  1    CASSPRTGRQY  11
                                     CA+ +RTGR+Y
CoxA6 & CoxA16                341    CAELARTGREY  351
```
RNA-dependent RNA polymerase Coxsackievirus A6 ALB73117.1
And polyprotein Coxsackievirus A16, AKN89892.1, 2072-2082

Uncharacterised protein Streptococcus pyogenes, QBX28410.1, 2-7,
And type 11 methyltransferase Streptococcus pneumoniae, CKH25746.1, 211-216,
And transcriptional regulatory protein Streptococcus pneumoniae, COF52299.1
```
S.pyogenes & S.pneumoniae                157   SPRTGR   162
                                       SPRTGR
TCR Heart 2.13                  2   ASSPRTGRQY  11
                                    A SP+TGR Y
S.pneumoniae                   36   APSPHTGRPY  45
```
hypothetical protein, Streptococcus pneumoniae, MDG7107425.1

transcriptional regulatory protein, Streptococcus pneumoniae, COF52299.1

```
TCR Heart 2.13                  4    SPRTGR---YE  11
                                     SPRTGR   YE
S.pneumoniae                  157    SPRTGRSILYE  167
```

collagen type VI alpha 6, partial, Homo sapiens, CAO81741.1

```
TCR Heart 2.13                  4    SPRTGRQY  11
                                     SPRTG+ Y
Collagen                      299    SPRTGKAY  306
```

capping protein regulator and myosin 1 linker 1, partial [Homo sapiens]
Sequence ID: KAI2541054.1Length: 932Number of Matches: 2

```
TCR Heart 2.13                  3    SSPRTGRQY  11
                                     SSP++GR+Y
Myosin 1 linker               750    SSPQAGRRY  758
```

F-actin-uncapping protein LRRC16A isoform X17, Homo sapiens, XP_054211808.1

```
TCR Heart 2.13                  3    SSPRTGRQY  11
                                     SSP++GR+Y
F-actin uncapping prot.      1130    SSPQAGRRY  1138
```

TCR Heart 2.14 CASSPRTGRYEQYF (1 Clone)

VP1, partial, Coxsackievirus A14, AAD40501.1

```
TCR Heart 2.14  1    CASSPRT  7
            CASS RT
CoxA14  121  CASSSRT  127
```

Membrane dipeptidase (Peptidase family M19), Streptococcus pneumoniae, CJF66504.1

```
TCR Heart 2.14  6    RTGRYEQY  13
            R GRYEQY
S.pneumoniae  268  RLGRYEQY  275
```

Carbohydrate diacid regulator, Streptococcus pneumoniae, COC58580.1

```
TCR Heart 2.14  8    GRYEQY  13
            GRYEQY
S.pneumoniae  168  GRYEQY  173
```

transcriptional regulatory protein, Streptococcus pneumoniae, COF52299.1

```
TCR Heart 2.14  4    SPRTGR---YE  11
            SPRTGR   YE
S.pneumoniae  157  SPRTGRSILYE  167
```

ABC transporter ATP-binding protein/permease, Streptococcus pyogenes, WP_011184891.1

```
TCR Heart 2.14  7    TGRYEQYF  14
            TGRYEQ F
S.pyogenes  226  TGRYEQEF  233
```

T-cell receptor beta chain junction region=TCR V beta 3 J beta 2.7 [human, inclusion body myositis patient 8, muscle-infiltrating lymphocytes, Peptide Partial, 23 aa] [Homo sapiens] AAB32012.1

```
Query  1   CASSPRTG-RYEQYF  14
           CASS R G  YEQYF
Sbjct  1   CASS-RQGLPYEQYF  14
O'Hanlon,T.P., Dalakas,M.C., Plotz,P.H. and Miller,F.W.
  TITLE     The alpha beta T-cell receptor repertoire in inclusion body
            myositis: diverse patterns of gene expression by
            muscle-infiltrating lymphocytes
  JOURNAL   J. Autoimmun. 7 (3), 321-333 (1994)
   PUBMED   7916906
```

TCR Heart 2.15   CASSQDVGQLTNYEQYF   (1 CLONE)

polyprotein, partial, Coxsackievirus B5, CCW33402.1

```
TCR Heart 2.15                  7     VGQLTNY  13
                                      VGQ+TNY
CoxB5                           1088  VGQVTNY  1094
```

polyprotein, partial, Coxsackievirus B5, CCW33402.1

```
TCR Heart 2.15                  2    ASSQ-DVGQL-TNYEQYF  17
                                     A SQ D  QL +N  QYF
CoxB5                           599  APSQSDQEQLFSN-VQYF  615
```

Uncharacterized conserved protein, Streptococcus pneumoniae, CKF69883.1

```
TCR Heart 2.15                  9    QLTNYEQY  16
                                     QLTNYEQY
S.pneumoniae                    115  QLTNYEQY  122
```

Uncharacterised protein, Streptococcus pneumoniae, CKH22399.1

```
TCR Heart 2.15                  11   TNYEQY  16
                                     TNYEQY
S.pneumoniae                    126  TNYEQY  131
```

threonine dehydratase, Streptococcus pneumoniae, VOI57995.1

```
TCR Heart 2.15                5    QDVGQLTNYE---QY  16
                                   Q VGQLT YE   QY
S.pneumoniae                  241  QKVGQLT-YEATRQY  254
```

putative PKS biosynthesis protein, Streptococcus pneumoniae, CJR91720.1

```
TCR Heart 2.15                2    ASSQDVGQLTN-YEQ  15
                                   AS Q+V QL++ YEQ
S.pneumoniae                  129  ASAQNVVQLDDVYEQ  143
```

T cell receptor beta chain, partial [Homo sapiens] ABR67593.1

```
Query  1   CASSQDVGQLTNYEQYF  17
           CASS DVG   T YEQYF
Sbjct  1   CASSLDVG--TAYEQYF  15
```

Menezes,C.A.S., Sullivan,A.K., Falta,M.T., Mack,D.G., Freed,B.M.,
Rocha,M.O.C., Gollob,K.J., Fontenot,A.P. and Dutra,W.O.
TITLE Oligoclonal Expansions among Specific V Expressing T Cells in Human Chagas Disease
JOURNAL Unpublished
https://www.ncbi.nlm.nih.gov/protein/ABR67593.1?report=genbank&log$=protalign&blast_rank=1&RID=G3M26FPY016 (accessed 13 Sep 2023)

T cell receptor beta chain, partial [Homo sapiens] ABR67597.1

```
Query  1   CASSQDVGQLTNYEQYF  17
           CASS D G  TNYEQYF
Sbjct  1   CASSLDSG--TNYEQYF  15
```

Menezes,C.A.S., Sullivan,A.K., Falta,M.T., Mack,D.G., Freed,B.M.,
Rocha,M.O.C., Gollob,K.J., Fontenot,A.P. and Dutra,W.O.
TITLE Oligoclonal Expansions among Specific V Expressing T Cells in Human
Chagas Disease
JOURNAL Unpublished
https://www.ncbi.nlm.nih.gov/protein/ABR67593.1?report=genbank&log$=protalign&blast_rank=1&RID=G3M26FPY016 (accessed 13 Sep 2023)

TCR Heart 2.16 CASSQGGAGAYEQYF (1 CLONE)

carboxymuconolactone decarboxylase family protein, Streptococcus anginosus, WP_003026283.1

```
TCR Heart 2.16                10   AYEQYF  15
                                   AYEQYF
S.angino.                     133  AYEQYF  138
```

D-alanyl-lipoteichoic acid biosynthesis protein DltD, Streptococcus pneumoniae, WP_061652184.1

```
TCR Heart 2.16                5    QGGAGAYEQYF  15
                                   QGGA+ ++QYF
S.pneumoniae                  101  QGGAASFNQYF  111
```

salivaricin A modification enzyme, Streptococcus pyogenes, VGW66486.1

```
TCR Heart 2.16                9   GAYEQY  14
                                  GAYEQY
S.pyogenes                    24  GAYEQY  29
```

actin related protein 2/3 complex subunit 1B, partial, Homo sapiens, KAI2546845.1

```
TCR Heart 2.16                2    ASSQGG--AGA  10
                                   ASS+GG  AGA
Actin-related protein         309  ASSEGGTAAGA  319
```

intermediate dynein chain, Homo sapiens, CAC17464.1
dynein axonemal heavy chain 9 isoform X4, Homo sapiens, XP_016879783.1, 2370-4

```
TCR Heart 2.16                11   YEQYF  15
                                   YE+YF
Dynein                        130  YEEYF  134
```

myosin-reactive immunoglobulin heavy chain variable region, partial [Homo sapiens] AAD56255.1
Sequence ID: Length: 125Number of Matches: 1

```
Query  1    CASSQGG  7
            CA SQGG
Sbjct  96   CARSQGG  102
```

T cell receptor beta chain, partial [Homo sapiens] ABR67596.1

```
Query  1   CASSQGGAGAYEQYF  15
           CASS  G/  YEQYF
Sbjct  1   CASSLDGGSSYEQYF  15
```

Menezes,C.A.S., Sullivan,A.K., Falta,M.T., Mack,D.G., Freed,B.M.,
Rocha,M.O.C., Gollob,K.J., Fontenot,A.P. and Dutra,W.O.
TITLE Oligoclonal Expansions among Specific V Expressing T Cells in Human Chagas Disease
JOURNAL Unpublished
https://www.ncbi.nlm.nih.gov/protein/ABR67593.1?report=genbank&log$=protalign&blast_rank=1&RID=G3M26FPY016 (accessed 13 Sep 2023)

TCR Heart 2.17 CASSQEPPGTRAYHDYEQYF (1 CLONE)

hypothetical protein, Streptococcus anginosus, WP_167702630.1

```
TCR Heart 2.17                  12  AYHDYEQY  19
                                    AYHD+EQY
S.anginosus                     31  AYHDHEQY  38
```

YtxH domain-containing protein, Streptococcus pneumoniae, WP_275584198.
Tn916 ORF15 signal peptide containing protein, Streptococcus pneumoniae, SNF13786.1

```
TCR Heart 2.17                  2    ASS-QEPPGTR--AYHDYEQ  18
                                     ASS QE P TR   YHD EQ
S.pneumoniae                    588  ASSVQERPTTRQEQYHD-EQ  606
```

Protein of uncharacterised function (DUF2515), Streptococcus pneumoniae, COE47240.1

```
TCR Heart 2.17                  9    GTRAYHDYEQY  19
                                     G+RA  DYEQY
S.pneumoniae                    253  GSRA--DYEQY  261
```

Enterotoxin 40 kDa subunit, Streptococcus pneumoniae, CJV90557.1

```
TCR Heart 2.17                  12  AYHDYEQY  19
                                    AY+DYE+Y
S.pneumoniae                    46  AYNDYEEY  53
```

hypothetical protein, Streptococcus anginosus, MCW1065389.1

```
TCR Heart 2.17                  7   PPGTRAYHD---YE  17
                                    PP +RAYHD   YE
S.anginosus                     20  PP-ARAYHDTVIYE  32
```

F-actin binding protein, isoform CRA_e, Homo sapiens, EAW60185.1

```
Actin-binding protein           279  SQEEPPG  285
                                     SQE PPG
TCR Heart 2.17                  4    SQEPPGT  10
                                      Q+PPGT
Actin-binding protein           39    QDPPGT  44
```

TCR Heart 2.18 CASSRSEREQYEQYF (1 CLONE)

hypothetical protein, Streptococcus sanguinis, WP_002895342.1

```
Sbjct     588  REEYEQY  594
               RE+YEQY
Query  5    RSEREQYEQY  14
            R+E EQY+
Sbjct  588  REEYEQYD  595
```

Phosphoribulokinase, Streptococcus anginosus, WP_270736522.1

```
Query  8    REQYEQYF  15
            REQYE Y+
Sbjct  164  REQYERYY  171
```

Phosphoribulokinase/Uridine kinase family protein, Streptococcus anginosus, ETI82303.1

```
Query  4   SRSEREQYEQYF  15
           SR  R+QYE+Y+
Sbjct  49  SR--RQQYEHYY  58
```

Myosin heavy chain, myosin-1, myosin-2, myosin-3, myosin-13, Homo sapiens, CAA31492.1NP_003793.2, CAA32167.1 ,AAD29948, XP_054172208.1
, NP_005954.3, AAI14546.1,EAW90006.1

```
Query  3     SSRSE----REQYEQ  13
             SSR +    REQYE+
Sbjct  1339  SSRHDCDLLREQYEE  1353
```

cardiac alpha-myosin heavy chain & myosin-6, Homo sapiens, CAA79675.1, NP_002462.2

```
Query  8     REQYEQ  13
             REQYE+
Sbjct  1346  REQYEE  1351
```

myosin IC, isoform CRA_a, Homo sapien, EAW90619.1

```
Query  6    SEREQYE---------QYF  15
            SE+E+YE         QYF
Sbjct  255  SEQEEYEAEGIAWEPVQYF  273
```

actin nucleation promoting factor, partial, Homo sapiens, ABX71502.1

```
Query  4    SRSEREQYEQ-----YF  15
            S S REQ EQ     YF
Sbjct  162  SISKREQLEQQVPENYF  178
```

dynein axonemal heavy chain 17 isoform X4, Homo sapiens, XP_024306782.1

```
Query  6     SEREQYEQYF  15
             S RE YE YF
Sbjct  2342  SPRELYELYF  2351
```

TCR Heart 2.19 CASSQDGGYTYEQYF (1 CLONE)

polyprotein, partial, Coxsackievirus B5, CCW33713.1

```
TCR Heart 2.19                   3    SSQDGGY  9
                                      SS+D+GY
CoxB5                            142  SSKDAGY  148
```

VP1, partial, Coxsackievirus A6, AFV68079.1

```
TCR Heart 2.19                   4   SQDGGYT  10
                                     SQD GYT
CoxA6                            46  SQD-GYT  51
```

capsid protein, partial, Coxsackievirus A8, QMU95405.1

```
TCR Heart 2.19                   5   QDGGYT---YE  12
                                     QDGG T   YE
CoxA8                            19  QDGG-TNKGYE  28
```

pyridine nucleotide-disulfide oxidoreductase, Streptococcus pneumoniae, COF02052.1

```
TCR Heart 2.19                   6   DGGYT-YEQ  13
                                     DGGYT YEQ
S.pneumoniae                     90  DGGYTAYEQ  98
```

Bacterial capsule synthesis protein PGA_cap, Streptococcus pneumoniae, CVY82333.1

```
TCR Heart 2.19                   3    SSQDGGYTYE  12
                                      S+Q+GGYT+E
S.pneumoniae                     347  SDQNGGYTFE  356
```

Uncharacterized protein conserved in bacteria, Streptococcus pneumoniae, CKF14060.1

```
TCR Heart 2.19                   9   YTYEQYF  15
                                     YTY+QYF
S.pneumoniae                     49  YTYSQYF  55
```

bacitracin transport ATP-binding protein BcrA, Streptococcus pneumoniae, CVN40703.1

```
TCR Heart 2.19                   8    GYTYEQ  13
                                      GYTYEQ
S.pneumoniae                     245  GYTYEQ  250
```

ABC transporter ATP-binding protein, Streptococcus pneumoniae, VTF11396.1

```
TCR Heart 2.19                   5    QDGGYTYE  12
                                      Q+GG+TYE
S.pneumoniae                     132  QEGGFTYE  139
```

myosin X, Homo sapiens, KAI2537022.1

```
TCR Heart 2.19                   4    SQDGGYTYEQ  13
                                      S DG Y+Y+Q
Myosin                           423  SPDGDYDYDQ  432
```

type X collagen, partial, Homo sapiens, AAA61221.1
```
TCR Heart 2.19                9   YTYEQY  14
                                  YTY++Y
Collagen                      77  YTYDEY  82
```

acetylcholine-specific T-cell receptor beta chain, partial [Homo sapiens] AAB25007.1
```
Query  1   CASSQDGGYTYEQYF  15
           CASS +G YT E +F
Sbjct  4   CASSLEG-YTGELFF  17
```

T cell receptor beta chain, partial [Homo sapiens] ABR67596.1
```
Query  1   CASSQDGGYTYEQYF  15
           CASS DGG +YEQYF
Sbjct  1   CASSLDGGSSYEQYF  15
```

Menezes,C.A.S., Sullivan,A.K., Falta,M.T., Mack,D.G., Freed,B.M., Rocha,M.O.C., Gollob,K.J., Fontenot,A.P. and Dutra,W.O.
TITLE Oligoclonal Expansions among Specific V Expressing T Cells in Human Chagas Disease
https://www.ncbi.nlm.nih.gov/protein/ABR67596.1?report=genbank&log$=protalign&blast_rank=1&RID=G47HCJ6X013 (accessed 13 Sep 2023)

T cell receptor beta chain, partial [Homo sapiens] ABR67594.1
Sequence ID: Length: 17Number of Matches: 1
```
Query  1   CASSQDGGYTYEQYF  15
           CASS D G TYEQYF
Sbjct  1   CASSLDQGTTYEQYF  15
```

Menezes,C.A.S., Sullivan,A.K., Falta,M.T., Mack,D.G., Freed,B.M., Rocha,M.O.C., Gollob,K.J., Fontenot,A.P. and Dutra,W.O.
TITLE Oligoclonal Expansions among Specific V Expressing T Cells in Human Chagas Disease
JOURNAL Unpublished
https://www.ncbi.nlm.nih.gov/protein/ABR67594.1?report=genbank&log$=protalign&blast_rank=9&RID=G47HCJ6X013 (accessed 13 Sep 2023)

T cell receptor beta chain, partial [Homo sapiens] ABR67598.1
```
Query  1   CASSQDGGYTYEQYF  15
           CASS D G  YEQYF
Sbjct  1   CASSLDSGTSYEQYF  15
```

Menezes,C.A.S., Sullivan,A.K., Falta,M.T., Mack,D.G., Freed,B.M., Rocha,M.O.C., Gollob,K.J., Fontenot,A.P. and Dutra,W.O.
TITLE Oligoclonal Expansions among Specific V Expressing T Cells in Human Chagas Disease
JOURNAL Unpublished
https://www.ncbi.nlm.nih.gov/protein/ABR67594.1?report=genbank&log$=protalign&blast_rank=9&RID=G47HCJ6X013 (accessed 13 Sep 2023)

TCR Heart 7.1 CASSPVEPNMTEAFFG (1 CLONE)

Nucleotidyl transferase of uncharacterised function (DUF1814), Streptococcus pneumoniae, CJD10567.1

```
TCR Heart 7.1                  9    NMTEAFFG  16
                                    NMTE FFG
S.pneumoniae                   303  NMTEMFFG  310
```

TrkH family potassium uptake protein, Streptococcus pneumoniae, MTV64622.1

```
TCR Heart 7.1                  8    PNMTEAFF  15
                                    PNM++AFF
S.pneumoniae                   24   PNMIDAFF  31
```

degenerate transposase, Streptococcus pyogenes, VGQ37628.1

```
TCR Heart 7.1                  10   MTEAFFG  16
                                    MTE FFG
S.pyogenes                     12   MTESFFG  18
```

ryanodine receptor 2 (cardiac), isoform CRA_b, Homo sapiens, EAW70070.1

```
Ryanodine rec.                3342      DMSEA  3346
                                        +M+EA
TCR Heart 7.1                  6     VEPNMTEAF  14
                                     VEP+M+ /F
Ryanodine rec.                 2447  VEPDMSAGF  2455
```

cardiac-enriched FHL2-interacting protein X2 (hypertrophy), Homo sapiens, XP_016871108.1

```
TCR Heart 7.1                    5     PVEPN  9
                                       PVEPN
Cardiac FHL2-interacting protein  284   PVEPN  288
```

myosin XIX, Homo sapiens, KAI2582507.1

```
TCR Heart 7.1                  3    SSPVEPNMTEAF  14
                                    SSPV   M EAF
Myosin                         41   SSPV---M-EAF  48
```

TCR Heart 7.2 CASSTLTGPTEAFFG (1 CLONE)

AMP-binding protein, partial;Streptococcus pneumoniae, WP_142998241.1Length

```
TCR Heart 7.2                  3    SSTLTGPTEAF  13
                                    S TLTGP++AF
S.pneumoniae                   32   SATLTGPSSAF  42
```

Putative transposase InsK for insertion sequence element IS150, Streptococcus agalactiae, AKI94966.1

```
TCR Heart 7.2                9   PTEAFFG  15
                                  PTE FFG
S.agalac.                    12   PTESFFG  18
```

hypothetical protein, partial, Streptococcus pneumoniae, WP_196301310.1
Sequence ID: Length: 77Number of Matches: 1

```
TCR Heart 7.2                3    SSTLTGP  9
                                  SSTLTGP
S.pneumoniae                 33   SSTLTGP  39
```

DnaD domain protein, Streptococcus sanguinis, WP_126435224.1

```
TCR Heart 7.2               4     STLTGPTEAFFG  15
                                  STLT   +E FFG
S.sanguinis                 96    STLT--AEEFFG  105
```

Chain A, Collagen alpha-1(XX) chain, Homo sapiens, 2EE3_A

```
TCR Heart 7.2                3    SSTLTG  8
                                  SSTLTG
Collagen                     86   SSTLTG  91
```

Collagen alpha-1(VII) chain isoform X7, Homo sapiens, XP_054201152.1

```
TCR Heart 7.2                6      LTGPTEA  12
                                    LTGPT A
Collagen                     2219   LTGPTGA  2225
```

myosin, heavy polypeptide 14, isoform CRA_a, Homo sapiens, EAW71838.1

```
TCR Heart 7.2                7    TGPTE  11
                                  TGPTE
Myosin                       43   TGPTE  47
```

cardiomyopathy associated 5, Homo sapiens, EAW95839.1

```
TCR Heart 7.2                1     CASSTL  6
                                   CASST+
Cardiomyopathy protein       252   CASSTM  257
```

TCR Heart 7.3 CASSQVLDNYGY (1 CLONE)

polyprotein, partial, Coxsackievirus A13, AGX00826.1

```
TCR Heart 7.3                1     CASSQV  6
                                   CAS+QV
CoxA13                       185   CASNQV  190
```

YfhO family protein, Streptococcus agalactiae, WP_150417292.1
Sequence ID: Length: 859Number of Matches: 1

```
TCR Heart 7.3                  1    CASSQVLD  8
                                    C SSQVLD
S.agalactiae                   527  CSSSQVLD  534
```

histidine--tRNA ligase, Streptococcus mutans, NLQ79294.1

```
TCR Heart 7.3                  4   SQVLDNYGY  12
                                   S +LDNYGY
S.mutans                       30  SRLLDNYGY  38
S.mutans                       20  SRLLDNYGY  28
```

ATP phosphoribosyltransferase regulatory subunit, Streptococcus mutans, WP_179957260.1

LCP family protein, Streptococcus anginosus, WP_195323471.1

```
TCR Heart 7.3                  5   QVLDNYGY  12
                                   QVL+N+GY
S.anginosus                    61  QVLENFGY  68
```

Protein of uncharacterised function (DUF2845), Streptococcus pneumoniae, CJL33301.1

```
TCR Heart 7.3                  6   QVLDNYGY  12
                                   +VLD+YGY
S.pneumoniae                   58  EVLDEYGY  65
```

single-stranded-DNA-specific exonuclease RecJ, Streptococcus pneumoniae, WP_057546754.1

```
TCR Heart 7.3                  4    SQVLDNY  10
                                    SQVL+NY
S.pneumoniae                   428  SQVLENY  434
S.pneumoniae                   4     QVLDNY  9
```

hypothetical protein, Streptococcus pneumoniae, WP_061630890.1

histidyl-tRNA synthetase, Streptococcus pneumoniae, CJK96554.1

```
TCR Heart 7.3                  5   QVLDNYGY  12
                                   Q+LD+YGY
S.pneumoniae                   31  QLLDSYGY  38
```

Myosin IE, Homo sapiens, KAI2574451.1

```
TCR Heart 7.3                  6    VLDNYGY  12
                                    VLD YG+
Myosin                         106  VLDIYGF  112
Myosin                         385  VLDIYGF  391
```

Myosin IB, isoform CRA_b, Homo sapiens, EAX10840.1

unconventional myosin-VIIb isoform X7, Homo sapiens, XP_054198142.1

```
TCR Heart 7.3                  4    SQVLD  8
                                    SQVLD
Myosin                         760  SQVLD  764
```

TCR Heart 7.4 CASSQDSGRVYSRYGY (1 CLONE)

polyprotein, partial, Coxsackievirus A17, AGF90750.1, AAF43589.1

```
TCR Heart 7.4                3   SSQDSGRVYSRYG  15
                                 S+QDSG +Y  YG
CoxA17                       19  STQDSGAAY--YG  29
CoxA17                       56  STQDSGAAY--YG  66
```

replication initiation factor domain-containing protein, Streptococcus agalactiae, WP_047205410.1

```
TCR Heart 7.4                10   VYSRYGY  16
                                  VYSRYGY
S.agalactae                  97   VYSRYGY  103
```

transcriptional regulator, Streptococcus pneumoniae, CWE88364.1

```
TCR Heart 7.4                9    RVYSRYGY  16
                                  R+YSRYGY
S.pneumoniae                 206  RAYSRYGY  213
```

helicase-exonuclease AddAB subunit AddA, Streptococcus sanguinis, WP_002906286.1

```
TCR Heart 7.4                10   VYSRYGY  16
                                  V+SRYGY
S.sanguinis                  133  VFSRYGY  139
```

ZmpA/ZmpB/ZmpC family metallo-endopeptidase, Streptococcus sanguinis, WP_268701444.1

```
TCR Heart 7.4                6     DSGRVYSRYGY  16
                                   DSGR Y+R GY
S.sanguinis                  2390  DSGR-YTRNGY  2399
```

Cardiomyopathy-associated protein 3 (Xin actin-binding protein 2) A4UGR9.2

```
TCR Heart 7.4                5     QDSGRVY  11
                                   Q+SGRV+
Cardiomyopathy protein       3310  QESGRVF  3316
```

Myosin Vc pre-powerstroke state, Homo sapiens, 5HMP_A

```
TCR Heart 7.4                11   YSRYG  15
                                  YSRYG
Myosin                       692  YSRYG  696
```

Collagen type XXVII alpha 1 chain, partial, Homo sapiens, KAI4008170.1

```
TCR Heart 7.4                4    SQDSGR  9
                                  SQDSGR
Collagen                     198  SQDSGR  203
```

Collagen-like cementum protein, Homo sapiens, ULE28343.1

```
TCR Heart 7.4                4    SQDSGRVYSR  13
                                  SQ +GRV SR
Cementum                     134  SQGTGRVSSR  143
```

Dynein cytoplasmic 1 intermediate chain 2, Homo sapiens, KAI2525768.1

```
TCR Heart 7.4                2   ASSQDSG  8
                                 A SQDSG
Dynein                       93  AGSQDSG  99
```

myosin-reactive immunoglobulin heavy chain variable region, partial [Homo sapiens] AAD56255.1

```
Query  1    CASSQDSGRV  10
            CA SQ  GR+
Sbjct  96   CARSQGGGRI  105
```

T cell receptor beta chain, partial [Homo sapiens] BAC01026.1

```
Query  1   CASSQDSGRVYSRYGY  16
           CASSQD G V   YGY
Sbjct  3   CASSQDRGQV---YGY  15
```

```
Saito,M., Higuchi,I., Saito,A., Izumo,S., Usuku,K., Bangham,C.R.
            and Osame,M.
  TITLE     Molecular analysis of T cell clonotypes in muscle-infiltrating
            lymphocytes from patients with human T lymphotropic virus type 1
            polymyositis
  JOURNAL   J. Infect. Dis. 186 (9), 1231-1241 (2002)
```

T cell receptor beta chain, partial [Homo sapiens] BAC01001.1

```
Query  1   CASSQDSGRVYSRYGY  16
           CASSQDSG      GY
Sbjct  3   CASSQDSG------GY  12
```

```
Saito,M., Higuchi,I., Saito,A., Izumo,S., Usuku,K., Bangham,C.R.
            and Osame,M.
  TITLE     Molecular analysis of T cell clonotypes in muscle-infiltrating
            lymphocytes from patients with human T lymphotropic virus type 1
            polymyositis
  JOURNAL   J. Infect. Dis. 186 (9), 1231-1241 (2002)
```

Heart 7.5 CASSQDKGAYFG (1 CLONE)

VP1 protein, partial, Coxsackievirus A18, CAD98894.1

```
CoxA18                          25    STQDTGAAYYG  35
                                      S+QD G AY+G
TCR Heart 7.5                   3     SSQDKG-AYFG  12
                                      SSQD G AY+G
CoxA13                          805   SSQDAGAAYYG  815
```

Polyprotein, Coxsackievirus A13, WAB71078.1

Polyprotein, Coxsackievirus A13, WAB71078.1

```
CoxA13                          6      SSQKVGA  12
                                       SSQ /GA
TCR Heart 7.5                   2     ASSQDKGA  9
                                      AS ++KG
CoxA13                          1336  ASLEEKG  1342
```

Polyprotein, Coxsackievirus B2, ANQ31694.1

```
TCR Heart 7.5                   6     DKGSYF  11
                                      DKG+YF
CoxB2                           2084  DKGEYF  2089
```

Polyprotein, Coxsackievirus B2, ANQ31694.1

```
TCR Heart 7.5                   3   SSQDKGA  9
                                    SSQ/\GA
CoxB2                           6   SSQKTGA  12
```

AAA family ATPase, Streptococcus anginosus, WP_220432070.1
AAA family ATPase, Streptococcus agalactiae, WP_199267360.1

```
TCR Heart 7.5                   3    SSQDK--GAYF  11
                                     SS+DK  GAYF
S.anginosus                     523  SSEDKRLGAYF  533
S.agalactiae                    510  SSEDKRMGAYF  520
```

ATPase family (AAA), Streptococcus pneumoniae, VSL20909.1

Dam family site-specific DNA-(adenine-N6)-methyltransferase, Streptococcus pneumoniae, WP_241003538.1

```
TCR Heart 7.5                   6    DKGAYF  11
                                     DKGAYF
S.pneumoniae                    655  DKGAYF  660
```

T-cell receptor beta chain, partial [Homo sapiens] BAC01027.1

```
Query  1   CASSQDKGA---YFG  12
           CASSQD GA   YFG
Sbjct  3   CASSQDSGADTQYFG  17
```

```
Saito,M., Higuchi,I., Saito,A., Izumo,S., Usuku,K., Bangham,C.R.
            and Osame,M.
  TITLE     Molecular analysis of T cell clonotypes in muscle-infiltrating
            lymphocytes from patients with human T lymphotropic virus type 1
            polymyositis
  JOURNAL   J. Infect. Dis. 186 (9), 1231-1241 (2002)
```

TCR Heart 7.6 CASSQDRGQGFNEQFFG (1 CLONE)

dephospho-CoA kinase, Streptococcus agalactiae, WP_079268457.1
```
TCR Heart 7.6                    6   DRGQGFNEQFF  16
                                      DR Q+F+EQFF
S.agalactiae                     66  DR-QAFSEQFF  75
```

Small%2C acid-soluble spore protein O, Streptococcus pneumoniae, CGF88855.1
```
TCR Heart 7.6                    2   ASSQDRGQGFNEQF  15
                                     AS Q  G G+NE+F
S.pneumoniae                     16  ASAQGQGAGYNEEF  29
```

hypothetical protein, partial, Streptococcus pneumoniae, WP_230681829.1
```
TCR Heart 7.6                    6   DRGQGFNEQF  15
                                     DRG+GFN QF
S.pneumoniae                     34  DRGSGFNRQF  43
```

IS1202 transposase, Streptococcus pneumoniae, VML57071.1
```
TCR Heart 7.6                    9   QGFNEQF  15
                                     Q+FNEQF
S.pneumoniae                     13  QAFNEQF  19
```

CMP-N-acetylneuraminate-beta-galactosamide-alpha-2%2C3-sialyltransferase, Streptococcus pneumoniae, CVP80484.1
```
TCR Heart 7.6                    12  NEQFFG  17
                                     NEQFFG
S.pneumoniae                     24  NEQFFG  29
```

unconventional myosin-VIIb isoform X8, Homo sapiens, XP_047300396.1
```
TCR Heart 7.6                    4    SQ----DRGQG  10
                                      SQ    DRGQG
Myosin                           259  SQVMASDRGQG  269
```

T cell receptor beta chain, partial [Homo sapiens] BAC01048.1
```
Query  1   CASSQDRGQGFNEQFFG  17
           CASS + GQGFNEQFFG
Sbjct  3   CASS-NPGQGFNEQFFG  18
```

```
Saito,M., Higuchi,I., Saito,A., Izumo,S., Usuku,K., Bangham,C.R.
            and Osame,M.
  TITLE     Molecular analysis of T cell clonotypes in muscle-infiltrating
            lymphocytes from patients with human T lymphotropic virus type 1
            polymyositis
  JOURNAL   J. Infect. Dis. 186 (9), 1231-1241 (2002)
```

T cell receptor beta chain, partial [Homo sapiens] BAC01001.1
```
Query  1   CASSQDRGQGFNEQFFG  17
           CASSQD G G+NEQFFG
Sbjct  3   CASSQDSG-GYNEQFFG  18
```

```
Saito,M., Higuchi,I., Saito,A., Izumo,S., Usuku,K., Bangham,C.R.
            and Osame,M.
  TITLE     Molecular analysis of T cell clonotypes in muscle-infiltrating
            lymphocytes from patients with human T lymphotropic virus type 1
```

```
            polymyositis
  JOURNAL   J. Infect. Dis. 186 (9), 1231-1241 (2002)
```

T cell receptor beta chain, partial [Homo sapiens] AAG15755.1

```
Query  1   CASSQDRGQGFNEQFFG  17
           CASS DRG  +NEQFFG
Sbjct  8   CASSPDRG--YNEQFFG  22

Slachta,C.A., Jeevanandam,V., Goldman,B., Lin,W.L. and
            Platsoucas,C.D.
  TITLE     Coronary arteries from human cardiac allografts with chronic
            rejection contain oligoclonal T cells: persistence of identical
            clonally expanded TCR transcripts from the early
            post-transplantation period (endomyocardial biopsies) to chronic
            rejection (coronary arteries)
  JOURNAL   J. Immunol. 165 (6), 3469-3483 (2000)
```

T cell receptor beta chain, partial [Homo sapiens] AAG15782.1

```
Query  1   CASSQDRGQGFNEQFFG  17
           CASS D GQG NEQFFG
Sbjct  8   CASSLDPGQGRNEQFFG  24

Slachta,C.A., Jeevanandam,V., Goldman,B., Lin,W.L. and
            Platsoucas,C.D.
  TITLE     Coronary arteries from human cardiac allografts with chronic
            rejection contain oligoclonal T cells: persistence of identical
            clonally expanded TCR transcripts from the early
            post-transplantation period (endomyocardial biopsies) to chronic
            rejection (coronary arteries)
  JOURNAL   J. Immunol. 165 (6), 3469-3483 (2000)
```

TCR Heart 7.7 CASSQNIGASGNEQFFG (1 CLONE)

polyprotein, partial, Coxsackievirus A1, WBR19182.1
polyprotein, partial, Coxsackievirus A19, WBR19366.1

```
TCR Heart 7.7                   5   QNIGASG  11
                                    QNI+ASG
CoxA1, CoxA19                   16  QNIAASG  22
```

polyprotein, partial, Coxsackievirus B4, AAK59941.1

```
TCR Heart 7.7                   2   ASSQNIGASGNEQFF  16
                                    A SQ    SG+EQ+F
CoxB4                           64  APSQ----SGQEQLF  74
```

Polyprotein, Coxsackievirus B2, ANQ31694.1

```
TCR Heart 7.7                   3   SSQNIG-------ASGN  12
                                    SSQ++G       ASGN
CoxB4                           6   SSQKTGAHETSLSASGN  22
```

H3 ,Streptococcus pneumoniae, CVM61464.1

```
TCR Heart 7.7                   2     ASSQNIGASGNEQ  14
                                      A S++I AS+NEQ
S.pneumoniae                    506   AGSEEISASANEQ  518
```

outer membrane biogenesis protein BamB, Streptococcus pneumoniae, CJR72959.1

```
TCR Heart 7.7                   4   SQNIGASGF  12
                                    SQN GASGF
S.pneumoniae                    47  SQNNGASGF  55
```

phospholipase D-like domain-containing protein, Streptococcus sanguinis, WP_004186649.1

```
TCR Heart 7.7                   3     SSQNIGASGFF  13
                                      SSQN G+SG+F
S.sanguinis                     312   SSQNFGTSGWF  322
```

thioester reductase domain-containing protein, Streptococcus sanguinis, WP_002924092.1

```
TCR Heart 7.7                   4     SQNI---GASGFFG  14
                                      SQNI   GA+GF G
S.sanguinis                     173   SQNIFMTGATGFLG  186
```

signal recognition particle-docking protein FtsY, Streptococcus pneumoniae, CVP51044.1

```
TCR Heart 7.7                   5     QNIGAS--GFF  13
                                      QNIGA   GFF
S.pneumoniae                    109   QNIGAGFRGFF  119
```

Quinol oxidase subunit 1, Streptococcus pneumoniae, COQ48439.1

```
TCR Heart 7.7                   9   ASGFFG  14
                                    ASGFFG
S.pneumoniae                    11  ASGFFG  16
```

Cardiomyopathy-associated protein 3 (xin actin-binding 2), Homo sapiens, KAI2525625.1

```
TCR Heart 7.7                  7      IGAS--GNEQFF  16
                                       I AS  GN QFF
Cardiomyopathy protein          1513   IDASERGNVQFF  1524
```

anti-acetylcholine receptor immunoglobulin kappa light chain variable region, partial [Homo sapiens] AAK61521.1

```
Query  1   CASSQNIGAS  10
           C \SQ+IG+S
Sbjct  23  CQASQDIGTS  32
```

T-cell receptor beta V6-2J1.4, TCRBV6-2J1.4 {complementarity determining region 3} [human, peripheral T-lymphocytes, insulin-dependent diabetes mellitus patient GAD65.05, Peptide Partial, 25 aa] [Homo sapiens] AAB46915.1

Sequence ID: Length: 25Number of Matches: 1

```
Query  1   CASSQNIG-ASGNEQFFG  17
           CASS    G A+G+E FFG
Sbjct  1   CASSPP-GRADGDELFFG  17
Weiss,U., Manfras,B.J., Terjung,D., Eiermann,T., Wolpl,A.,
            Loliger,C., Kuhnl,P. and Boehm,B.O.
  TITLE     In vitro stimulation with glutamic acid decarboxylase (GAD65)
leads to an oligoclonal response of peripheral T-cells in an IDDM patient
  JOURNAL   Scand. J. Immunol. 42 (6), 673-678 (1995)
```

TCR Heart 7.8 CASSQDWQNCYFG (1 CLONE)

Polyprotein, Coxsackievirus B5, QQL13745.1

```
TCR Heart 7.8                    2    ASSQDWQNC  10
                                      A+S+DWQNC
CoxB5                            874  ATSHDWQNC  882
                                      A+S DWQNC
CoxB3 & CoxB4                    874  ATSADWQNC  882
```

polyprotein , Coxsackievirus B3, AAV34212.1

polyprotein , Coxsackievirus B4, QKZ25342.1

polyprotein , Echovirus E11, WIL60493.1

```
TCR Heart 7.8                    5    QDWQNC  10
                                      QDWQNC
Ecovirus E11                     887  QDWQNC  892
```

MFS transporter, Streptococcus sanguinis, WP_176798328.1

```
TCR Heart 7.8                    3    SSQDWQ  8
                                      SSQDWQ
S.sanguinis                      198  SSQDWQ  203
```

Domain of uncharacterised function (DU1801), Streptococcus pneumoniae, CVY52767.1

```
TCR Heart 7.8                    4   SQDWQNCY  11
                                     SQ WQ+CY
S.pneumoniae                     17  SQ-WQDCY  23
```

SidA/IucD/PvdA family monooxygenase, Streptococcus pneumoniae, MXQ49484.1

```
TCR Heart 7.8                4    SQDWQNCY  11
                                  SQ+WQ CY
S.pneumoniae                 117  SQEWQ-CY  123
```

cholinergic receptor nicotinic delta subunit, Homo sapiens, KAI2527445.1

```
Acetylcholine receptor       161  DWQNC  165
                                  DWQNC
TCR Heart 7.8                6    DWQNC  10
                                  DWQNC
Acetylcholine receptor       28   DWQNC  32
```

acetylcholine receptor epsilon subunit, extracellular domain, Homo sapiens, AAB46913.1

unconventional myosin-XVI isoform X5, Homo sapiens, XP_054230245.1

```
TCR Heart 7.8                1     CASSQDWQN  8
                                   C + QDWQ+
Myosin                       1194  CEEGQDWQS  1201
```

T cell receptor beta chain, partial [Homo sapiens] ABR67605.1

```
Query  1   CASSQDW---QNCYFG  13
           CASSQD    Q  YFG
Sbjct  2   CASSQDSTDTQ--YFG  15
Menezes,C.A.S., Sullivan,A.K., Falta,M.T., Mack,D.G., Freed,B.M.,
            Rocha,M.O.C., Gollob,K.J., Fontenot,A.P. and Dutra,W.O.
  TITLE     Oligoclonal Expansions among Specific V Expressing T Cells in
Human Chagas Disease
  JOURNAL   Unpublished
```

TCR Heart 7.9 CASSYGYEQYF   (1 CLONE)

IS3 family transposase, Streptococcus pneumoniae, WP_088849926.1

```
TCR Heart 7.9                   5    YGYEQYF  11
                                     YGYEQ F
S.pneumoniae                    404  YGYEQNF  410
```

isochorismatase family protein, Streptococcus dysgalactiae, WP_192942422.1

```
TCR Heart 7.9                   5    YGYEQYF  11
                                     YGY+QYF
S. pneum.                       142  YGYDQYF  148
```

T cell receptor beta chain variable region, partial [Homo sapiens] AAW33959.1

```
Query  1   CASS--YG-YEQYF  11
           CASS  YG YEQYF
Sbjct  38  CASSLTYGPYEQYF  51
```

```
Ellis,N.M., Li,Y., Hildebrand,W., Fischetti,V.A. and
            Cunningham,M.W.
  TITLE     T cell mimicry and epitope specificity of cross-reactive T cell
            clones from rheumatic heart disease
  JOURNAL   J. Immunol. 175 (8), 5448-5456 (2005)
```

TCR Heart 7.10 CASSQGAEPLQYEQYF   (1 CLONE)

VP1, partial, Coxsackievirus A6, WCB86769.1

```
TCR Heart 7.10                  10   LQYEQ  14
                                     LQYEQ
CoxA6                           112  LQYEQ  116
```

polyprotein, partial, Coxsackievirus A1, WBR19198.1

```
TCR Heart 7.10                  9    PLQYEQ  14
                                     PLQY+Q
CoxA1                           303  PLQYSQ  308
```

phage protein, Streptococcus pyogenes, VGT93048.1

```
TCR Heart 7.10                  9   PLQYEQYF  16
                                    PLQY QYF
S.pyogenes                      7   PLQY-QYF  13
```

aquaporin Z, Streptococcus pneumoniae, COI98568.1

```
TCR Heart 7.10                  9    PLQYEQYF  16
                                     PL YEQYF
S.pneumoniae                    263  PL-YEQYF  269
```

phosphoribosylaminoimidazole synthetase, Streptococcus pneumoniae, CWH83385.1

```
TCR Heart 7.10                  5    QGAEPLQYEQYF  16
                                     QGAEPL    YF
S.pneumoniae                    95   QGAEPL----YF  102
```

exonuclease domain-containing protein, Streptococcus pyogenes, WP_227868769.1

```
TCR Heart 7.10                  9    PLQYEQY  15
                                     PLQYE+Y
S.pyogenes                      323  PLQYEDY  329
```

Exodeoxyribonuclease V beta chain, Streptococcus pneumoniae, CVQ41851.1

```
TCR Heart 7.10                  8     EPLQYEQ  14
                                      EPLQ+EQ
S.pneumoniae                    1091  EPLQFEQ  1097
```

C-5 cytosine-specific DNA methylase protein, Streptococcus pneumoniae NP127, EHE51824.1

```
TCR Heart 7.10                  1    CASSQGAEPLQY-EQ  14
                                     C SSQG   +QY EQ
S.pneumoniae                    86   CPSSQG---MQYSEQ  9
```

Myosin IC, isoform CRA_a, Homo sapiens, EAW90619.1

```
TCR Heart 7.10                  6    GAEPLQY  12
                                     G+EP+QY
Myosin                          714  GSEPIQY  720
```

Myosin MYO9A, partial, Homo sapiens, SPT35772.1

```
Myosin                          1326  SSQGSLELLSYEE  1338
                                      SSQG+ E L+YE+
TCR Heart 7.10                  3     SSQGA-EPLQYEQ  14
                                      SSQG+ E L+YE+
Myosin                          1267  SSQGSLELLSYEE  1279
```

unconventional myosin-IXa isoform X15, Homo sapiens, XP_047288533.1

collagen alpha-1(II) chain isoform X4 [Homo sapiens] XP_016874320.1

```
Query  6      GAEPLQY  12
              G +PLQY
Sbjct  1050   GPDPLQY  1056

Query  4     SQGAEPLQ  11
             S GA+ LQ
Sbjct  527   SPGAQGLQ  534
```

collagen alpha-1(II) chain isoform X4 [Homo sapiens] XP_016874320.1

```
Query  6      GAEPLQY  12
              G +PLQY
Sbjct  1050   GPDPLQY  1056

Query  4     SQGAEPLQ  11
             S GA+ LQ
Sbjct  527   SPGAQGLQ  534
```


laminin A chain, partial [Homo sapiens] CAA41418.1

```
Query  3     SSQGAE--PLQ-YEQY  15
             S+Q AE   LQ YE+Y
Sbjct  620   STQ-AEGLSLQPYEEY  634
```

dynein axonemal heavy chain 9 isoform X4 [Homo sapiens] XP_016879783.1

```
Query  12    YEQYF  16
             YE+YF
Sbjct  2370  YEHYF  2374

Query  1   CASSQGAEPL  10
           CA S  AE L
Sbjct  46  CAGSAEAEQL  55
```

TCR Heart 7.11 CASSQDKNYEQYF (1 CLONE)

M protein, Streptococcus pyogenes, WP_128888759.1

```
TCR Heart 7.11                  4   SQDKNYEQY  12
                                    S++ NYEQY
S.pyogenes                      73  SHNNNYEQY  81
```

bifunctional metallophosphatase/5'-nucleotidase, Streptococcus agalactiae, WP_000726931.1

```
TCR Heart 7.11                  7    KNYEQY  12
                                     KNYEQY
S.agalagiae                     218  KNYEQY  223
```

dynein axonemal intermediate chain 7 isoform X11,Homo sapiens, XP_047285034.1

```
TCR Heart 7.11                  5    QDKNYEQY  12
                                     QDKN  QY
Dynein                          158  QDKNIIQY  165
```

TCR Heart 7.12 CASSFNREAYEQYF (1 CLONE)

anthranilate synthase component I, Streptococcus pneumoniae, CJK74717.1

```
TCR Heart 7.12                  3    SSFNREAYEQ  12
                                     SSF+RE YEQ
S.pneumoniae                    219  SSFSREDYEQ  228
```

hypothetical protein, Streptococcus sanguinis, WP_149565477.1

```
TCR Heart 7.12                  3   SSFNREAY--EQY  13
                                    SSF+RE+Y  +QY
S.sanguinis                     84  SSFSRESYTTQQY  96
```

DnaB-like helicase C-terminal domain-containing protein, Streptococcus pneumoniae, WP_078712880.1

```
TCR Heart 7.12                  3    SSFNREAYE  11
                                     SSFNR+ YE
S.pneumoniae                    563  SSFNRDNYE  571
```

DNA cytosine methyltransferase, partial, Streptococcus pneumoniae, WP_168970539.1

```
TCR Heart 7.12                  8   EAYEQYF  14
                                    EAYEQ+F
S.pneumoniae                    10  EAYEQFF  16
```

dynein, cytoplasmic 1, intermediate chain 1, isoform CRA_b, Homo sapiens, EAW76752.1

```
TCR Heart 7.12                  4    SFNREAYEQ  12
                                     SFNR+ Y++
Dynein                          223  SFNRQFYDE  231
```

dynein axonemal heavy chain 17 isoform X4, Homo sapiens, XP_024306782.1

```
TCR Heart 7.12                  7     REAYEQYF  14
                                      RE+YE YF
Dynein                          2344  RELYELYF  2351
```

T cell receptor beta chain, partial [Homo sapiens] AAG15792.1

```
Query  1   CASSFNREAYEQYF  14
           CASSF+R +YEQYF
Sbjct  8   CASSFRR-GYEQYF  20
```

```
Slachta,C.A., Jeevanandam,V., Goldman,B., Lin,W.L. and
            Platsoucas,C.D.
  TITLE     Coronary arteries from human cardiac allografts with chronic
            rejection contain oligoclonal T cells: persistence of identical
            clonally expanded TCR transcripts from the early
            post-transplantation period (endomyocardial biopsies) to chronic
            rejection (coronary arteries)
  JOURNAL   J. Immunol. 165 (6), 3469-3483 (2000)
```

TCR Heart 7.13 CASSSGLAGYEQYF

VP1, partial, Coxsackievirus A12, QWM97587.1
```
CoxA12                          34   SRSGLAG  40
                                     S SGLAG
TCR Heart 7.13                  3    SSSGLAG  9
                                     S SGLAG
CoxA5                           8    SRSGLAG  14
```
capsid protein, partial, Coxsackievirus A5, QMU95395.1

Polyprotein, Coxsackievirus B1, P08291
```
TCR Heart 7.13                  2    ASSSGL  7
                                     ASS+GL
CoxB1                       599      ASSQGL  604
```

Dipeptidase, Streptococcus agalactiae 138P, AHN30202.1
```
TCR Heart 7.13                  8     AGYEQYF  14
                                      AGYEQYF
S.agalactiae                    281   AGYEQYF  287
```

DNA repair protein RecO, Streptococcus pneumoniae, CJD30205.1
```
TCR Heart 7.13                  6     GLAGYEQYF  14
                                      GL G++QYF
S.pneumoniae                    130   GLSGFDQYF  138
```

carbohydrate ABC transporter permease, Streptococcus pneumoniae, MTW00257.1
```
TCR Heart 7.13                  1    CASSSGLAGY  10
                                     CA+SS LAGY
S.pneumoniae                    71   CATSS-LAGY  79
```

membrane protein BUG1, Streptococcus pneumoniae, CJC53609.1
```
S.pneumoniae                    246   GLSSYDQYF  254
                                      GL  Y+QYF
TCR Heart 7.13                  6     GLAGYEQYF
                              GL  Y+QYF
                                      GL  Y+QYF
S.pneumoniae                    583   GLSSYDQYF  591
```
Putative membrane protein ydgH, Streptococcus pneumoniae, CJO25202.1

collagen alpha-3(IV) chain isoform X6, Homo sapiens, XP_054196411.1
```
TCR Heart 7.13                  1     CASSSGLAG  9
                                      CA+S GL G
Collagen                        422   CAGSPGLPG  430
```

T cell receptor beta chain, partial [Homo sapiens] BAC01046.1
```
Query  1   CASSSGLAG-YEQYF  12
           CASS+GLAG  +QYF
Sbjct  3   CASSAGLAGGVTQYF  17
```

```
Saito,M., Higuchi,I., Saito,A., Izumo,S., Usuku,K., Bangham,C.R.
            and Osame,M.
  TITLE     Molecular analysis of T cell clonotypes in muscle-infiltrating
            lymphocytes from patients with human T lymphotropic virus type 1
            polymyositis
  JOURNAL   J. Infect. Dis. 186 (9), 1231-1241 (2002)
```

```
T cell receptor beta chain, partial [Homo sapiens] ABR67600.1
Query  1   CASSSGLAG--YEQYF  12
           CASSSGLAG   EQ+F
Sbjct  1   CASSSGLAGVSDEQFF  16
Menezes,C.A.S., Sullivan,A.K., Falta,M.T., Mack,D.G., Freed,B.M.,
            Rocha,M.O.C., Gollob,K.J., Fontenot,A.P. and Dutra,W.O.
  TITLE     Oligoclonal Expansions among Specific V Expressing T Cells in
Human  Chagas Disease
  JOURNAL   Unpublished
```

https://www.ncbi.nlm.nih.gov/protein/ABR67600.1?report=genbank&log$=protalign&blast_rank=6&RID=G3Z8ZE1D016 (accessed 13 Sep 2023)

TCR Heart 7.14 CASSQDRGQYEQYF (1 CLONE)

Chain F, The heavy chain of fab 9B5, Coxsackievirus A16, 7YMS_F

```
TCR Heart 7.14                 1    CASSQDRGQYEQYF  14
                                    CA    RG+YE+YF
CoxA16                         96   CA----RGDYENYF  105
```

6S rRNA methyltransferase GidB, Streptococcus pneumoniae, VSH22310.1

```
TCR Heart 7.14                 3   SSQDRGQYEQYF  14
                                   SSQ++ Q+E YF
S.pneumoniae                   18  SSQQKEQFERYF  29
```

Uncharacterized protein conserved in bacteria, Streptococcus pneumoniae, CJS00925.1

```
TCR Heart 7.14                 6    DRGQYEQY  13
                                    D +QYEQY
S.pneumoniae                   215  DQAQYEQY  222
```

hypothetical protein, Streptococcus pyogenes, AJW83682.1

```
TCR Heart 7.14                 3   SSQDRGQY----EQY  13
                                   SS+DR++Y    EQY
S.pyogenes                     22  SSEDRANYQALKEQY  36
```

Cardiac peroxidase, Homo sapiens, AAX70929.1

```
TCR Heart 7.14                 2    ASSQDRGQYE  11
                                    A ++D GQYE
Cardiac peroxidase             478  AAQHDQGQYE  487

TCR Heart 7.14                 1     CASSQDRGQY  10
                                     CA++ +RGQ+
Cardiac peroxidase             1299  CADCRSRGQF  1308
```

T cell receptor beta chain, partial [Homo sapiens] BAC01026.1

```
Query  1   CASSQDRGQQY  11
           CASSQDRGQ Y
Sbjct  3   CASSQDRGQVY  13

Saito,M., Higuchi,I., Saito,A., Izumo,S., Usuku,K., Bangham,C.R.
            and Osame,M.
  TITLE     Molecular analysis of T cell clonotypes in muscle-infiltrating
            lymphocytes from patients with human T lymphotropic virus type 1
            polymyositis
  JOURNAL   J. Infect. Dis. 186 (9), 1231-1241 (2002)
```

TCR Heart 7.15 CASSQDLQGRYEQYF

Polyprotein, Coxsackievirus B2, Q9YLG5

```
TCR Heart 7-15:                  3    SSQDLQG    9
                                      SSQ+ QG
CoxB2                          676    SSQTSQG    682
```

Polyprotein, Coxsackievirus B1, SPS68029.1

```
TCR Heart 7.15                 2    ASSQDL   7
                                    ASSQ L
CoxB1                          329  ASSQGL   334
```

Polyprotein, Coxsackievirus A9, P21404

```
TCR Heart 7-15                   4   SQD-–LQG    9
                                     SQD  LQG
CoxA9                          675   SQDNKLQG     683
```

transposase, partial, Streptococcus sanguinis, WP_196757391.1

```
TCR Heart 7.15                 4   SQDLQGRYEQY  14
                                   SQDL  RYE Y
S.sanguinis                    29  SQDLRNRYELY  39
```

chloride channel protein, Streptococcus pyogenes, WP_136111792.1

```
TCR Heart 7.15                 7     LQGRYE-QY  14
                                     LQGRYE QY
S.pyogenes                     230   LQGRYEIQY  238
```

GNAT family N-acetyltransferase, partial, Streptococcus pneumoniae, WP_142325397.1
Sequence ID: Length: 142Number of Matches: 1

```
TCR Heart 7.15                 4     SQDLQGRYEQY  14
                                     SQDLQG +EY+
S.pneumoniae                   131   SQDLQGNFESY  141
```

Glycosyltransferase, Streptococcus pneumoniae, WP_235977465.1

```
TCR Heart 7.15                 7    LQGR-Y-EQYF  15
                                    LQGR Y EQYF
S.pneumoniae                   84   LQGRMYIEQYF  94
```

FtsK/SpoIIIE domain-containing protein, Streptococcus anginosus, WP_229045148.1

```
TCR Heart 7.15                 10    RYEQYF  15
                                     RYEQYF
S.aginosus                     190   RYEQYF  195
```

myosin-9, Homo sapiens, NP_002464.1

```
TCR Heart 7.15                 6      DLQGRYEQ  13
                                      DLQGR EQ
Myosin                         1572   DLQGRDEQ  1579
```

POTE-2 alpha-actin, Homo sapiens, ABP57734.1

```
TCR Heart 7.15                 3     SSQDLQG---RQY  12
                                     SSQDL+G   R+Y
Actin                          332   SSQDLSGQTAREY  344
```

TCR Heart 7.16 CASSQDKNYEQYF (1 CLONE)

NADH-quinone oxidoreductase subunit L, Streptococcus pneumoniae, COR59093.1

```
TCR Heart 7.16                 6     DKNYEQYF  13
                                     DKNY QYF
S.pneumoniae                   140   DKNYRQYF  147
```

M protein,Streptococcus pyogenes, WP_128888759.1

```
TCR Heart 7.16                 4    SQDKNYEQY  12
                                    S++ NYEQY
S.pyogenes                     73   SHNNNYEQY  81
```

bifunctional metallophosphatase/5'-nucleotidase, Streptococcus agalactiae, WP_000726933.1

```
TCR Heart 7.16                 7     KNYEQY  12
                                     KNYEQY
S.agalactiae                   218   KNYEQY  223
```

dynein axonemal intermediate chain 7 isoform X11, Homo sapiens, XP_047285034.1

```
TCR Heart 7.16                  5    QDKNYEQY  12
                                     QDKN  QY
Dynein                          158  QDKNIIQY  165
```

T cell receptor beta chain, partial [Homo sapiens] ABR67597.1

```
Query  1   CASSQD--KNYEQYF  13
           CASS D   NYEQYF
Sbjct  1   CASSLDSGTNYEQYF  15
```

Menezes,C.A.S., Sullivan,A.K., Falta,M.T., Mack,D.G., Freed,B.M.,
Rocha,M.O.C., Gollob,K.J., Fontenot,A.P. and Dutra,W.O.
TITLE Oligoclonal Expansions among Specific V Expressing T Cells in Human Chagas Disease
JOURNAL Unpublished
https://www.ncbi.nlm.nih.gov/protein/ABR67597.1?report=genbank&log$=protalign&blast_rank=19&RID=G429G2GA013 (accessed 13 Sep 2023)
